# Deep Brain Stimulation in Parkinson Disease: A Meta-analysis of the Long-term Neuropsychological Outcomes

**DOI:** 10.1007/s11065-022-09540-9

**Published:** 2022-03-23

**Authors:** Madalina Bucur, Costanza Papagno

**Affiliations:** Center for Mind/Brain Sciences (CIMeC), University of Trento, Trento, Italy

**Keywords:** Parkinson’s disease (PD), Deep brain stimulation (DBS), Memory, Language, Executive functions, GRADE

## Abstract

Deep brain stimulation (DBS) of the subthalamic nucleus (STN) or globus pallidum internus (GPi) improves motor functions in patients with Parkinson’s disease (PD) but may cause a decline in specific cognitive domains. The aim of this systematic review and meta-analysis was to assess the long-term (1–3 years) effects of STN or GPi DBS on four cognitive functions: (i) memory (delayed recall, working memory, immediate recall), (ii) executive functions including inhibition control (Color-Word Stroop test) and flexibility (phonemic verbal fluency), (iii) language (semantic verbal fluency), and (iv) mood (anxiety and depression). Medline and Web of Science were searched, and studies published before July 2021 investigating long-term changes in PD patients following DBS were included. Random-effects model meta-analyses were performed using the R software to estimate the standardized mean difference (SMD) computed as Hedges’ g with 95% CI. 2522 publications were identified, 48 of which satisfied the inclusion criteria. Fourteen meta-analyses were performed including 2039 adults with a clinical diagnosis of PD undergoing DBS surgery and 271 PD controls. Our findings add new information to the existing literature by demonstrating that, at a long follow-up interval (1–3 years), both positive effects, such as a mild improvement in anxiety and depression (STN, Hedges’ g = 0,34, p = 0,02), and negative effects, such as a decrease of long-term memory (Hedges’ g = -0,40, p = 0,02), verbal fluency such as phonemic fluency (Hedges’ g = -0,56, p < 0,0001), and specific subdomains of executive functions such as Color–Word Stroop test (Hedges’ g = -0,45, p = 0,003) were observed. The level of evidence as qualified with GRADE varied from low for the pre- verses post-analysis to medium when compared to a control group.

**Supplementary Information:**

The online version contains supplementary material available at 10.1007/s11065-022-09540-9.

## Introduction

Parkinson’s disease (PD) is a progressive neurodegenerative condition characterized by three motor symptoms including rigidity, resting tremor, and bradykinesia. However, there is increasing evidence that PD patients can suffer several non-motor disturbances, such as sleep disorders, cognitive impairment, and behavioral changes, that can appear early and dominate the clinical picture during progression. The relationship between PD and behavioral disturbances was explained by the connection between the basal ganglia ventral region and the cingulate and orbital cortices, and the connection between the basal ganglia medial region and the orbitofrontal and prefrontal areas (Papagno & Trojano, 2018; Trojano & Papagno, 2018).

Opportunities to use technology to modulate or influence brain circuitry and human behavior have increased in recent years, with deep brain stimulation (DBS) being the most important and accepted treatment (Marsili et al., 2021). Targets for DBS are chosen based on their predominant symptomatology. Neurostimulation of the subthalamic nucleus (STN) allows a reduction of levodopa intake in advanced PD (Deuschl et al., 2006), while globus pallidum internus (GPi) stimulation seems to reduce dyskinesia (Krause, 2001; Zhang et al., 2021) and psychiatric symptoms (Bang Henriksen et al., 2016). Analyzing the subthalamic versus pallidal DBS therapeutic efficacy for PD, Elgebaly et al. (2018) concluded that there were no significant differences between the two procedures. Specifically, both improve motor function and, consequently, daily living (Deuschl et al., 2006). Only psychomotor processing speed, as measured by the Stroop color-naming test, seemed to favor the GPi DBS group (Elgebaly et al., 2018).

Even though DBS (specifically [STN] or [GPi] stimulation) was first FDA approved for PD in 2003, few studies have reported a follow-up greater than five years (Hitti et al., 2020). Among them, Bang Henriksen et al. (2016) reported the survival rate and outcomes, such as presence of hallucinations, dementia, and nursing home placement, of PD patients treated with DBS with at least ten years of follow-up. They observed that dementia was present in 46% and hallucinations in 58% of the 79 PD patients. Furthermore, older age at surgery was correlated with an increased prevalence of nursing home placement. Other outcome domains such as patient satisfaction, motor symptom control, and ability to perform activities of daily living (ADLs) were instead investigated by Hitti et al. (2020) who reported an overall improvement. Nevertheless, after a very long follow-up, more than 20 years post DBS, different ethical questions might emerge, such as the ones addressed by Gilbert and Lancelot (2021). They reported a case study and analyzed how extending life span without improving quality of life may result in a burden for patients and families.

Another domain that can be affected in PD is mood. Two randomized prospective studies reported no change in depression between baseline and after six months in a class I trial, while anxiety seemed to improve (Kurtis et al., 2017; Witt et al., 2008). The authors suggest caution in the interpretation of such changes after DBS because the Beck anxiety inventory, used in the evaluation, included several items with a strong somatic connection.

An important concern about DBS is how it affects cognitive performance. Indeed, exclusion criteria include dementia or a significant dysexecutive syndrome and depression or anxiety. This is accurate for studies, but in a clinical setting, cognitive impairment, depression, and similar disorders are considered relative neurobehavioral contraindications to DBS. Therefore, it is crucial to investigate cognitive domains, considering that decline could occur independently as a consequence of the pathological process and of aging.

A number of publications (Kurtis et al., 2017; Parsons et al., 2006; Witt et al., 2013) reported that STN DBS produces a statistically significant but mild decrease in executive functions and working memory. However, no significant cognitive changes were reported in 57% of the studies included in a meta-analysis (Appleby et al., 2007). Of note is that these review conclusions are based on DBS across a variety of conditions, not just PD. Recent reviews of cognitive outcomes after DBS in PD found heterogeneous cognitive effects, although deficits in verbal fluency were consistent and related to micro-lesions (Cernera et al., 2019, 2020; Mulders et al., 2021). In these meta-analyses and reviews, different neuropsychological instruments are used to investigate the same function, but tests investigating the same cognitive domain can in fact involve different components. Moreover, time of testing after DBS is not taken into account. The time of testing, measured as the length of time between DBS and neuropsychological evaluation, can have an important impact on the cognitive domain. At three months after DBS the patient may not be completely recovered (micro-lesions) which may affect performance, and considering that DBS is a long-term stimulation therapy, from our perspective it is important to understand what occurs one year or more after the DBS, as some effects might emerge over time. A too-long interval could possibly include effects of ageing and of disease progression.

Considering these issues, we performed a more careful selection of studies to avoid possible pitfalls (Funkiewiez, 2004). The primary aim of the current review was to provide a comprehensive overview and meta-analysis of the DBS long-term effects on four cognitive functions, namely, memory, executive functions, language, and mood, as measured by specific neuropsychological tests.

This review focuses on i) specific domains that are believed to be affected by DBS based on the previous literature, ii) specific neuropsychological tests (i.e., those used most in the selected papers to obtain more precise information); iii) a well-defined period (12 to 36 months after DBS), and iv) explicit avoidance of the insertion of data from the same clinical population more than once, as some studies are based on overlapping or identical samples.

## Methods

The present meta-analysis, conducted in accordance with the Preferred Reporting Items for Systematic Reviews and Meta-Analyses (PRISMA) guidelines (Liberati et al., 2009; Moher et al., 2015), is based on 48 studies (Table 1) investigating the long-term (12 to 36 months) cognitive changes in PD patients following DBS. Four cognitive domains were considered: (i) memory, namely, delayed recall, working memory (backward digit span), and immediate recall, (ii) executive functions, namely, inhibition control (color-word Stroop test) and flexibility (phonemic verbal fluency), (iii) language (semantic verbal fluency), and (iv) mood (anxiety and depression).

**Table 1:** Summary of the studies’ characteristics

**Nr.**	**Study**	**Country of origin**	**Design**	**Target Area**	**DBS Implantation**	**Levels of Evidence Modified Sackett Scale**	**Prospective /Retrospective**	**Participant’s recruitment**	**Conclusions**
1.	Pillon, B., Ardouin, C., Damier, P., Krack, P., Houeto, J. L., Klinger, H., & Agid, Y. (2000). Neuropsychological changes between “off” and “on” STN or GPi stimulation in Parkinson’s disease. Neurology, 55 (3), 411–418	France	within (pre vs. post)	STN or GPi	bilateral	4	not specified	not specified	no cognitive deficit at 12 months, except for lexical fluency
2.	Dujardin, K., Defebvre, L., Krystkowiak, P., Blond, S., & Destee, A. (2001). Influence of chronic bilateral stimulation of the subthalamic nucleus on cognitive function in Parkinson's disease. Journal of neurology, 248(7), 603–611	France	within (pre vs. post)	STN	bilateral	4	not specified	consecutively recruited	in some patients it can induce overall cognitive decline or behavioral changes
3.	Woods, S. P., Fields, J. A., Lyons, K. E., Koller, W. C., Wilkinson, S. B., Pahwa, R., & Tröster, A. I. (2001). Neuropsychological and quality of life changes following unilateral thalamic deep brain stimulation in Parkinson's disease: a one-year follow-up. Acta neurochirurgica, 143 (12), 1273–1278	USA	within (pre vs. post)	STN	unilateral (5 left, 1 right)	4	prospective	consecutively recruited	long-term neurocognitive safety and QOL improvements following DBS
4.	Daniele, A., Albanese, A., Contarino, M. F., Zinzi, P., Barbier, A., Gasparini, F., & Scerrati, M. (2003). Cognitive and behavioural effects of chronic stimulation of the subthalamic nucleus in patients with Parkinson’s disease. J Neurol Neurosurg Psychiatry, 74 (2), 175–182	Italy	ABBA design within (pre vs. post)	STN	bilateral	4	not specified	not specified	DBS does not per se appear to impair cognitive performance in patients with PD and may alleviate the postoperative decline in verbal fluency
5.	Moretti, R., Torre, P., Antonello, R. M., Capus, L., Marsala, S. Z., Cattaruzza, T., & Bava, A. (2003). Neuropsychological changes after subthalamic nucleus stimulation: a 12 month follow-up in nine patients with Parkinson's disease. Parkinsonism & Related Disorders, 10 (2), 73–79	Italy	between (DBS VS control) and within (pre vs. post)	STN	bilateral	3	prospective	not specified	a slowing of cognitive activity, with a reduction of quantitative production, but with an increase in control of linguistic production
6.	Funkiewiez, A., Ardouin, C., Caputo, E., Krack, P., Fraix, V., Klinger, H., & Pollak, P. (2004). Long term effects of bilateral subthalamic nucleus stimulation on cognitive function, mood, and behaviour in Parkinson’s disease. Journal of Neurology, Neurosurgery & Psychiatry, 75(6), 834–839	France	within (pre vs. post)	STN	bilateral	4	retrospective	consecutively recruited	STN stimulation did not lead to global cognitive deterioration
7.	Smeding, H. M., Esselink, R. A., Schmand, B., Koning-Haanstra, M., Nijhuis, I., Wijnalda, E. M., & Speelman, J. D. (2005). Unilateral pallidotomy versus bilateral subthalamic nucleus stimulation in PD. Journal of neurology, 252 (2), 176–182	Netherlands	within (pre vs. post)	GPi	unilateral (4 left, 10 right)	4	prospective	not specified	bilateral STN stimulation has slightly more negative effects on executive functioning than unilateral pallidotomy
8.	Castelli, L., Perozzo, P., Zibetti, M., Crivelli, B., Morabito, U., Lanotte, M., & Lopiano, L. (2006). Chronic deep brain stimulation of the subthalamic nucleus for Parkinson’s disease: effects on cognition, mood, anxiety and personality traits.European neurology, 55(3), 136–144	Italy	within (pre vs. post)	STN	bilateral	4	prospective	consecutively recruited	STN DBS is cognitively safe since the only relevant change observed was a mild decrease in verbal fluency tasks
9.	Cilia, R., Siri, C., Marotta, G., De Gaspari, D., Landi, A., Mariani, C. B., & Antonini, A. (2007). Brain networks underlining verbal fluency decline during STN-DBS in Parkinson's disease: an ECD-SPECT study. Parkinsonism & related disorders, 13(5), 290–294	Italy	between (DBS VS control) and within (pre vs. post)	STN	bilateral	3	prospective	not specified	category fluency tasks selectively declined after STN-DBS independently form dose decrements of dopaminergic medications
10.	Contarino, M. F., Daniele, A., Sibilia, A. H., Romito, L. M., Bentivoglio, A. R., Gainotti, G., & Albanese, A. (2007). Cognitive outcome 5 years after bilateral chronic stimulation of subthalamic nucleus in patients with Parkinson’s disease. Journal of Neurology, Neurosurgery & Psychiatry, 78(3), 248–252	Italy	within (pre vs. post)	STN	bilateral	4	not specified	not specified	STN DBS is associated with a low cognitive and behavioral morbidity over a 5-year follow-up
11.	Klempirova, O., Jech, R., Urgosik, D., Klempir, J., Spackova, N., Roth, J., & Ruzicka, E. (2007). Deep brain stimulation of the subthalamic nucleus and cognitive functions in Parkinson’s disease. Prague Medical Report, 108(4), 315–323	Czech Republic	within (pre vs. post)	STN	bilateral	4	not specified	not specified	patients treated by STN DBS tend to worsen in executive functions and in logical memory
12.	Ory‐Magne, F., Brefel‐Courbon, C., Simonetta‐Moreau, M., Fabre, N., Lotterie, J. A., Chaynes, P., & Rascol, O. (2007). Does ageing influence deep brain stimulation outcomes in Parkinson's disease? *Movement disorders: official journal of the Movement Disorder Society*, *22*(10), 1457–1463	France	within (pre vs. post)	STN	bilateral (2 unilateral)	4	prospective	consecutively recruited	cognitive impairment showed no correlation, but apathy and depression were positively correlated with age
13.	Rothlind, J. C., Cockshott, R. W., Starr, P. A., & Marks, W. J. (2007). Neuropsychological performance following staged bilateral pallidal or subthalamic nucleus deep brain stimulation for Parkinson's disease. Journal of the International Neuropsychological Society, 13(1), 68–79	USA	within (pre vs. post)	STN or GPi	bilateral 15 m unilateral 6 m	4	not specified	not specified	DBS is associated with small reductions in speeded information processing and working memory
14.	Witjas, T., Kaphan, E., Régis, J., Jouve, E., Chérif, A. A., Péragut, J. C., & Azulay, J. P. (2007). Effects of chronic subthalamic stimulation on nonmotor fluctuations in Parkinson's disease. Movement disorders: official journal of the Movement Disorder Society, 22(12), 1729–1734	France	within (pre vs. post)	STN	bilateral	4	prospective	consecutively recruited	STN DBS alleviates non-motor fluctuations
15.	Fraraccio, M., Ptito, A., Sadikot, A., Panisset, M., & Dagher, A. (2008). Absence of cognitive deficits following deep brain stimulation of the subthalamic nucleus for the treatment of Parkinson's disease. Archives of clinical neuropsychology, 23(4), 399–408	France	within (pre vs. post)	STN	bilateral	4	not specified	not specified	there was no deterioration on any cognitive test as a result of DBS
16.	Heo, J. H., Lee, K. M., Paek, S. H., Kim, M. J., Lee, J. Y., Kim, J. Y., & Jeon, B. S. (2008). The effects of bilateral subthalamic nucleus deep brain stimulation (STN DBS) on cognition in Parkinson disease. Journal of the neurological sciences, 273(1–2), 19–24	Republic of Korea	within (pre vs. post)	STN	bilateral	4	not specified	not specified	STN DBS might have minor detrimental long-term impacts on memory and frontal lobe function
17.	Zangaglia, R., Pacchetti, C., Pasotti, C., Mancini, F., Servello, D., Sinforiani, E.,& Nappi, G. (2009). Deep brain stimulation and cognitive functions in Parkinson's disease: A three‐year controlled study. Movement disorders: official journal of the Movement Disorder Society, 24(11), 1621–1628	Italy	between (DBS VS control) and within (pre vs. post)	STN	bilateral	3	prospective	consecutively recruited	a worsening of verbal fluency after DBS, but the short-term worsening of frontal-executive functions was found to be transient
18.	Castelli, L., Rizzi, L., Zibetti, M., Angrisano, S., Lanotte, M., & Lopiano, L. (2010). Neuropsychological changes 1-year after subthalamic DBS in PD patients: a prospective controlled study. Parkinsonism & Related Disorders, 16(2), 115–118	Italy	between (DBS VS control) and within (pre vs. post)	STN	bilateral	3	prospective	consecutively recruited	phonemic verbal fluency declined one year after STN DBS, while the other cognitive domains did not change significantly
19.	Follett, K. A., Weaver, F. M., Stern, M., Hur, K., Harris, C. L., Luo, P., & Pahwa, R. (2010). Pallidal versus subthalamic deep-brain stimulation for Parkinson's disease. New England Journal of Medicine, 362(22), 2077–2091	USA	within (pre vs. post)	STN or GPi	bilateral	4	not specified	not specified	- patients undergoing STN required a lower dose of dopaminergic agents than did those undergoing GPi – visuomotor processing speed declined more after STN - the level of depression worsened after STN and improved after GPi
20.	Kishore, A., Rao, R., Krishnan, S., Panikar, D., Sarma, G., Sivasanakaran, M. P., & Sarma, S. (2010). Long‐term stability of effects of subthalamic stimulation in Parkinson's disease: Indian Experience. Movement disorders, 25(14), 2438–2444	India	within (pre vs. post)	STN	bilateral	4	prospective	consecutively recruited	global scores for mood and cognition did not show significant worsening
21.	Mikos, A., Zahodne, L., Okun, M. S., Foote, K., & Bowers, D. (2010). Cognitive declines after unilateral deep brain stimulation surgery in Parkinson's disease: a controlled study using Reliable Change, part II. The Clinical Neuropsychologist, 24(2), 235–245	USA	between (DBS VS control) and within (pre vs. post)	STN or GPi	unilateral	3	not specified	not specified	a small proportion of DBS patients demonstrate reliable decline in the executive functioning tests
22.	Smeding, H. M., Speelman, J. D., Huizenga, H. M., Schuurman, P. R., & Schmand, B. (2011). Predictors of cognitive and psychosocial outcome after STN DBS in Parkinson's disease. Journal of Neurology, Neurosurgery & Psychiatry, 82 (7), 754–760	Netherlands	between (DBS VS control) and within (pre vs. post)	STN	bilateral	3	prospective	consecutively recruited	STN DBS improves quality of life; but a profile of cognitive decline can be found in a significant number of patients
23.	Williams, A. E., Arzola, G. M., Strutt, A. M., Simpson, R., Jankovic, J., & York, M. K. (2011). Cognitive outcome and reliable change indices two years following bilateral subthalamic nucleus deep brain stimulation. Parkinsonism & related disorders, 17(5), 321–327	USA	between (DBS VS control) and within (pre vs. post)	STN	bilateral	3	not specified	consecutively recruited	impairments in nonverbal recall, oral information processing speed, and lexical and semantic fluency in STN DBS patients compared to PD controls 2 years post-surgery
24.	Zibetti, M., Merola, A., Rizzi, L., Ricchi, V., Angrisano, S., Azzaro, C., & Rizzone, M. (2011). Beyond nine years of continuous subthalamic nucleus deep brain stimulation in Parkinson's disease. Movement Disorders, 26(13), 2327–2334	Italy	within (pre vs. post)	STN	bilateral	4	prospective	consecutively recruited	4 patients (29%) developed a significant cognitive decline over the follow-up period
25.	Sjöberg, R. L., Lidman, E., Häggström, B., Hariz, M. I., Linder, J., Fredricks, A., & Blomstedt, P. (2012). Verbal fluency in patients receiving bilateral versus left-sided deep brain stimulation of the subthalamic nucleus for Parkinson's disease. Journal of the International Neuropsychological Society, 18(3), 606–611	Sweden	within (pre vs. post)	STN	unilateral (left-sided) or bilateral	4	not specified	consecutively recruited	unilateral STN DBS of the speech dominant hemisphere is associated with significantly less declines in measures of verbal fluency as compared to bilateral stimulation
26.	Yamanaka, T., Ishii, F., Umemura, A., Miyata, M., Horiba, M., Oka, Y., & Ojika, K. (2012). Temporary deterioration of executive function after subthalamic deep brain stimulation in Parkinson's disease. Clinical neurology and neurosurgery, 114(4), 347–351	Japan	within (pre vs. post)	STN	bilateral	4	not specified	not specified	temporary deterioration of executive function may occur in the short term after STN DBS, whereas motor function is usually improved
27.	Kim, H. J., Jeon, B. S., Yun, J. Y., Kim, Y. E., Yang, H. J., & Paek, S. H. (2013). Initial cognitive dip after subthalamic deep brain stimulation in Parkinson disease. Journal of neurology, 260(8), 2130–2133	Germany	within (pre vs. post)	STN	bilateral	4	not specified	not specified	decline in global cognitive function was faster in the first 6 months after surgery, compared with that after 6 months
28.	Schuepbach, W. M. M., Rau, J., Knudsen, K., Volkmann, J., Krack, P., Timmermann, L., & Deuschl, G. (2013). Neurostimulation for Parkinson's disease with early motor complications. New England Journal of Medicine, 368(7), 610–622	Germany & France	between RCT (DBS VS control) and within (pre vs. post)	STN	bilateral	1 b	not specified	not specified	Neurostimulation was superior to medical therapy alone at a relatively early stage of Parkinson’s disease, before the appearance of severe disabling motor complications
29.	Asahi, T., Nakamichi, N., Takaiwa, A., Kashiwazaki, D., Koh, M., Dougu, N., & Kuroda, S. (2014). Impact of bilateral subthalamic stimulation on motor/cognitive functions in Parkinson’s disease. Neurologia medico-chirurgica, oa-2013	Japan	within (pre vs. post)	STN	bilateral	4	prospective	not specified	bilateral STN DBS did not affect any score on cognitive examinations
30.	Janssen, M. L., Duits, A. A., Tourai, A. M., Ackermans, L., Leentjes, A. F., van Kranen-Mastenbroek, V., & Temel, Y. (2014). Subthalamic nucleus high-frequency stimulation for advanced Parkinson's disease: motor and neuropsychological outcome after 10 years. Stereotactic and functional neurosurgery, 92(6), 381–387	Germany	within (pre vs. post)	STN	bilateral	4	prospective	not specified	- memory function seemed to improve in the short term, but there was a significant decline between 1 and 5 years after surgery - mood remained relatively stable during follow-up, - one third of the patients showed impulsive behavior after surgery
31.	Merola, A., Rizzi, L., Artusi, C. A., Zibetti, M., Rizzone, M. G., Romagnolo, A., & Lopiano, L. (2014). Subthalamic deep brain stimulation: clinical and neuropsychological outcomes in mild cognitive impaired parkinsonian patients. Journal of neurology, 261(9), 1745–1751	Italy	within (pre vs. post)	STN	bilateral	4	prospective	not specified	- long-lasting efficacy of STN DBS on motor functions in both PD-MCI and normal cognition subjects - PD-MCI patients showed a more precocious cognitive impairment
32.	Rizzone, M. G., Fasano, A., Daniele, A., Zibetti, M., Merola, A., Rizzi, L., & Albanese, A. (2014). Long-term outcome of subthalamic nucleus DBS in Parkinson's disease: from the advanced phase towards the late stage of the disease?. Parkinsonism & related disorders, 20(4), 376–381	Italy	within (pre vs. post)	STN	bilateral	4	not specified	consecutively recruited	confirms the long-term safety and efficacy of STN DBS in PD
33.	Jiang, L. L., Liu, J. L., Fu, X. L., Xian, W. B., Gu, J., Liu, Y. M., & Pei, Z. (2015). Long-term efficacy of Subthalamic nucleus deep brain stimulation in Parkinson's disease: a 5-year follow-up study in China. Chinese medical journal, 128(18), 2433	China	within (pre vs. post)	STN	bilateral	4	not specified	consecutively recruited	STN DBS is an effective intervention for PD, patients required lower voltage and medication for satisfactory symptom control
34.	Odekerken, V. J., Boel, J. A., Geurtsen, G. J., Schmand, B. A., Dekker, I. P., de Haan, R. J., & NSTAPS Study Group. (2015). Neuropsychological outcome after deep brain stimulation for Parkinson disease. Neurology, 84(13), 1355–1361	Germany	within (pre vs. post)	STN or GPi	bilateral	4	prospective	not specified	no clinically significant differences in neuropsychological outcome between GPi DBS and STN DBS
35.	Tang, V., Zhu, C. X., Chan, D., Lau, C., Chan, A., Mok, V., & Poon, W. S. (2015). Evidence of improved immediate verbal memory and diminished category fluency following STN-DBS in Chinese-Cantonese patients with idiopathic Parkinson’s disease. Neurological Sciences, 36(8), 1371–1377	China	within (pre vs. post)	STN	bilateral	4	prospective	not specified	- slightly negative effects of STN DBS on verbal fluency; - improvement of immediate verbal memory
36.	Tramontana, M. G., Molinari, A. L., Konrad, P. E., Davis, T. L., Wylie, S. A., Neimat, J. S., & Salomon, R. M. (2015). Neuropsychological effects of deep brain stimulation in subjects with early stage Parkinson's disease in a randomized clinical trial. Journal of Parkinson's disease, 5(1), 151–163	USA	between RCT (DBS VS control) and within (pre vs. post)	STN	bilateral	1b	prospective	not specified	DBS group: modest reductions on a few measures of attention, executive function, and word fluency at 12 months; the differences were largely diminished at 24 months
37	Boel, J. A., Odekerken, V. J., Schmand, B. A., Geurtsen, G. J., Cath, D. C., Figee, M., & van Laar, T. (2016). Cognitive and psychiatric outcome 3 years after globus pallidus pars interna or subthalamic nucleus deep brain stimulation for Parkinson's disease. Parkinsonism & related disorders, 33, 90–95	Germany	within (pre vs. post)	STN or GPi	bilateral	4	prospective	not specified	no significant between-group differences were found on the MDRS, neuropsychological tests, and psychiatric questionnaires
38.	Tröster, A. I., Jankovic, J., Tagliati, M., Peichel, D., & Okun, M. S. (2017). Neuropsychological outcomes from constant current deep brain stimulation for Parkinson's disease. Movement Disorders, 32(3), 433–440	USA	within (pre vs. post)	STN	bilateral	3	prospective	not specified	within-group analyses revealed declines in category and switching verbal fluency in both groups, but only the stimulation group had letter verbal fluency and Stroop task declines
39.	Foki, T., Hitzl, D., Pirker, W., Novak, K., Pusswald, G., & Lehrner, J. (2018). Individual cognitive change after DBS-surgery in parkinson’s disease patients using reliable change index methodology. Neuropsychiatrie, 32(3), 149–158	Austria	between (DBS VS control) and within (pre vs. post)	STN	bilateral	3	prospective	consecutively recruited	there was cognitive change in individual patients, but the change was very heterogeneous with gains and losses
40.	Acera, M., Molano, A., Tijero, B., Bilbao, G., Lambarri, I., Villoria, R., & Gomez-Esteban, J. C. (2019). Long-term impact of subthalamic stimulation on cognitive function in patients with advanced Parkinson's disease. Neurología (English Edition), 34(9), 573–581	Spain	within (pre vs. post)	STN	bilateral	4	prospective	not specified	- significant impairment of verbal function, - improvements in motor function are less pronounced at 5 years - performance in activities of daily living improves, lowering the required doses of antiparkinsonian drugs
41.	Liu, F. T., Lang, L. Q., Yang, Y. J., Zhao, J., Feng, R., Hu, J., & Wu, J. J. (2019). Predictors to quality of life improvements after subthalamic stimulation in Parkinson’s disease. Acta neurologica Scandinavica, 139(4), 346–352	China	within (pre vs. post)	STN	bilateral	4	prospective	not specified	- 51.1% of the patients reported a better QOL, - cognition and bodily discomfort improved significantly after the surgery
42.	Pusswald, G., Wiesbauer, P., Pirker, W., Novak, K., Foki, T., & Lehrner, J. (2019). Depression, quality of life, activities of daily living, and subjective memory after deep brain stimulation in Parkinson disease—A reliable change index analysis. International journal of geriatric psychiatry, 34(11), 1698–1705	Austria	between (DBS VS control) and within (pre vs. post)	STN	bilateral	3	prospective	not specified	after DBS—mental health, depressive symptoms, and physical health benefit most, while the domains activities of daily living and subjective memory functioning are rather constant
43.	Dietrich, A. D., Koeppen, J. A., Buhmann, C., Pötter-Nerger, M., Pinnschmidt, H. O., Oehlwein, C., & Gulberti, A. (2020). Sex Disparities in the Self-Evaluation of Subthalamic Deep Brain Stimulation Effects on Mood and Personality in Parkinson's Disease Patients. Frontiers in neurology, 11	Germany	within (pre vs. post)	STN	bilateral	4	prospective	consecutively recruited	the BDI-I score was not significantly modulated by the factor DBS time, but it was modulated by sex: female patients had significantly higher BDI scores than male patients
44.	Jost, S. T., Ray Chaudhuri, K., Ashkan, K., Loehrer, P. A., Silverdale, M., Rizos, A., & Dafsari, H. S. (2021). Subthalamic Stimulation Improves Quality of Sleep in Parkinson Disease: A 36-Month Controlled Study. Journal of Parkinson's Disease, (Preprint), 1–13	UK & Germany	between (DBS VS control) and within (pre vs. post)	STN	bilateral	3	prospective	not specified	report beneficial effects of STN-DBS on quality of sleep at 36-month follow-up, which were associated with QOL improvement independent of depression and dopaminergic medication
45.	Leimbach, F., Atkinson-Clement, C., Wilkinson, L., Cheung, C., & Jahanshahi, M. (2020). Dissociable effects of subthalamic nucleus deep brain stimulation surgery and acute stimulation on verbal fluency in Parkinson’s disease. Behavioural brain research, 388, 112,621	UK	between (DBS VS control) and within (pre vs. post)	STN	bilateral	3	not specified	not specified	the STN-DBS effect on VF are a surgical and not an acute STN stimulation effects
46.	Mulders, A. E., Temel, Y., Tonge, M., Schaper, F. L., van Kranen-Mastenbroek, V., Ackermans, L., … & Duits, A. (2021). The association between surgical characteristics and cognitive decline following deep brain stimulation of the subthalamic nucleus in Parkinson’s disease. Clinical Neurology and Neurosurgery, 200, 106,341	Netherlands	within (pre vs. post)	STN	bilateral	4	prospective	not specified	the electrode passage through the right prefrontal lobe may contribute to subtle changes in executive function
47.	You, Z., Wu, Y. Y., Wu, R., Xu, Z. X., Wu, X., & Wang, X. P. (2020). Efforts of subthalamic nucleus deep brain stimulation on cognitive spectrum: From explicit to implicit changes in the patients with Parkinson's disease for 1 year. CNS neuroscience & therapeutics, 26(9), 972–980	China	between (DBS VS control) and within (pre vs. post)	STN	bilateral	3	not specified	not specified	STN-DBS as a neuromodulatory tool in the Chinese PD population not only improves motor symptoms but also cognitive function to a certain extent, such as the decline of executive function and verbal fluency
48.	Volonté, M. A., Clarizio, G., Galantucci, S., Scamarcia, P. G., Cardamone, R., Barzaghi, L. R., & Filippi, M. (2021). Long term follow-up in advanced Parkinson’s disease treated with DBS of the subthalamic nucleus. Journal of Neurology, 1–10	Italy	within (pre vs. post)	STN	bilateral	3	retrospective and prospective	not specified	STN-DBS had no long-lasting effect on axial symptoms and cognitive functions

*STN* subthalamic nucleus, *GPi* internal globus pallidus, *DBS* deep brain stimulation, *RCT* randomized control trial, *QOL* quality of life, *MCI* mild cognitive impairment, *MDRS* Mattis Dementia Rating Scale, *PD* Parkinson's disease

### Literature Search and Study Selection

Two electronic databases including MEDLINE (https://pubmed.ncbi.nlm.nih.gov) and Web of Science were searched for studies investigating DBS and its impact on the previously mentioned cognitive domains in PD patients. The considered papers were published between January 2000 and June 2021. The following terms were used in our research: (1) “Parkinson’s disease” or “PD” AND (2) “deep brain stimulation” or “DBS” AND (3) “cognition”, “memory”, “executive functions”, “language”, “depression”, “anxiety”. The reference lists of included studies and relevant reviews were searched to identify additional studies (Altinel et al., 2019; Appleby et al., 2007; Barbosa & Charchat-Fichman, 2019; Büttner et al., 2019; Cernera et al., 2020; Combs et al., 2015; Constantinescu et al., 2017).

Titles, abstracts, and full-text articles were screened independently by the authors and evaluated for eligibility based on the following inclusion and exclusion criteria:

#### Inclusion Criteria

- interventions designed for adults with advanced Parkinson's disease,
- DBS stimulation (unilateral or bilateral) of STN or GPi,
- DBS specified as main intervention or treatment,
- outcomes were measurable continuous variables including at least one of the four areas (memory, executive functions, language, mood),
- neuropsychological data were reported before and after DBS surgery, or between PD DBS and control group (PD without DBS),
- at least one standardized neuropsychological instrument from the following was used: delayed recall, backward digit span, immediate recall, color-word Stroop test, phonemic verbal fluency, semantic verbal fluency, anxiety, and depression scales,
- the follow-up period was between 12 and 36 months. We considered that a period of 12 months could be long enough to exclude the effects of the surgical procedure, while a period longer than 36 months could include changes in cognitive functions due to ageing or progression of the neurodegenerative disease (and not due to DBS),
- at least 5 participants in the study,
- peer-reviewed publications,
- published in English,

When several papers were derived from the same study, either with increased recruitment or extended follow-up evaluations, we chose the one with the higher number of participants and the most complete data reported at follow-up. If papers deriving from the same study reported results for different neuropsychological tests, then all papers were included in this review but in different meta-analysis, making sure that only one outcome from the same population was included each time.

#### Exclusion Criteria

- other cephalic stimulation sites, for example, caudal zona incerta (Philipson et al., 2020),
- a follow-up period less than 12 months or more than 36 months,
- pathologies other than PD,
- case reports and research studies with fewer than five participants,
- articles from gray literature (i.e., literature that is not formally published in sources such as books or journal articles, e.g., unpublished Ph.D. thesis),
- We chose not to include Ph.D. theses for two main reasons. Often, Ph.D. students embargo their dissertations (i.e., for 2 to 3 years they are not accessible even upon request), and good theses are published in journals as research articles.
- studies not published in, nor translated into, English,
- data could not be extracted because the study lacked data integrity to analyze treatment effects and no reply was obtained when writing to the authors.

As far as we know, there is no consensus regarding which tests or scales are to be used by clinicians nor which cognitive functions are to be evaluated in PD patients undergoing DBS (Papagno & Trojano, 2018; Trojano & Papagno, 2018). Therefore, we chose tests that were reported more often in the literature and that better capture the functions of interest (Dujardin et al., 2016). Specifically, we created a database with more than 50 different neuropsychological tests and selected those most frequently used. As already reported, these tests were: (i) delayed recall, backward digit span (working memory), and immediate recall to measure MEMORY, (ii) the color–word Stroop test and phonemic verbal fluency to measure EXECUTIVE FUNCTIONS, (iii) semantic verbal fluency for LANGUAGE, (iv) anxiety and depression scales to measure MOOD. In this last case, different but equivalent scales were used. Similarly, different delayed and immediate recall tests were employed to reach the highest statistical power (i.e., a higher number of included papers). In the results section, we reported which tests were used in each included publication. Most of the data were based on verbal memory tasks, which, from our perspective, does not mean that all the tests are equivalent but indicates that most of the instruments share a common objective.

We ran a preliminary selection based on title, keywords, and abstract excluding those that clearly did not satisfy our criteria. Subsequently, we made a further selection by inspecting the full manuscripts and applying the inclusion and exclusion criteria. Unresolved papers were discussed by the authors to reach a consensus.

### Data Extraction

For each included paper, the relevant information to be extracted concerned i) intervention characteristics, including the target area, unilateral or bilateral DBS implantation (Table 1), ii) study characteristics, including design, language, main objective, and conclusions (Table 1), and iii) patient characteristics including sample size (treatment and control group), gender, age, education, disease duration, time from DBS, before and after DBS levodopa equivalent daily dosage (LEDD), Unified Parkinson's Disease Rating Scale (UPDRS) Part III: clinician-scored motor evaluation (before and after DBS) (Table 2).

**Table 2:** Summary of patients’ characteristics

**Id**	**Study**	**Number at** **Follow-up**	**Gender**		**Age** **(Years)**	**Education** **(Years)**	**Disease Duration** **(Years)**	**Time from the DBS** **(Months)**	**LEDD mg/day**		**UPDRS-III**	
			**M**	**F**					**Before DBS**	**After DBS**	**pre-DBS**	**post DBS**
1.	Pillon et al. (2000)	56	33	23	54.1 (7)	12.7(3.8)	15.6 (4.1)	3 & **12**	STN: 1110 (570) GPi: 744 (264)	STN: 348 (292) GPi:873 (478)	STN: 49.8 (14.8) GPi: 49.8 (14.8)	N.S
2.	Dujardin et al. (2001)	9 of 11	6	3	54.78 (8.15)	11.67 (2.83)	13.11 (2.93)	3 & **12**	1525 (534)	1051 (527)	21.66 (4.88)	7.50 (2.74)
3.	Woods et al. (2001)	6	5	1	70.83 (8.75)	13 (3.52)	7.33 (2.66)	**12**	N.S	N.S	23.60 (11.55)	23 (10.99)
4.	Daniele et al. (2003)	20	11	9	57.0 (7.8)	11.8 (5.2)	14.2 (5.4)	3. 6 & **12**	1395.8 (644.1)	500.7 (328.8)	24.0 (9.0)	22.1 (12.3)
5.	Moretti et al. (2003)	18	6	3	68.7 (7.89)	11.34 (5.67)	8.91 (2.34)	1. 6 & **12**	1432	668	61.72	26.32 (6.26)
6.	Funkiewiez (2004)	77 of 84	43	34	55 (8)	N.S	15 (5)	**12** & 24	N.S	N.S	N.S	N.S
7.	Smeding et al. (2005)	14	5	9	62.1 (8.1)	10.2 (3.1)	11 (7–20)	6 & **12**	1260 (340–2614)	1110 (410–2904)	N.S	N.S
8.	Castelli et al. (2006)	72 of 79	38	27	60.5 (6.5)	8.7 (4.2)	15.1 (5.1)	**15** (12–20)	1.010.5 (419.9)	447.1 (284.8)	54.4 (13.4)	27.2 (11.7)
9.	Cilia et al. (2007)	20	14	6	59.1 (7.4)	N.S	13.2 (3.1)	**12**	951 (465)	316 (295)	14.8 (8.1)	10.4 (6.1)
10.	Contarino et al. (2007)	26 of 41	7	4	57.5 (7.4)	11.0 (5.1)	15.5 (5.6)	**12** & 60	1406.55 (750.15)	427.66 (354.89)	24.27 (8.68)	20.82 (12.11)
11.	Klempírová et al. (2007)	19	13	6	57.4 (6)	N.S	14.9 (4)	**11—14**	769 (341)	510 (270)	N.S	N.S
12.	Ory‐Magne et al. (2007)	45 of 47	26	19	60.1 (8.7)	N.S	13.5 (3.6)	**12**. 24	1466 (665)	595 (437)	18.1 (8.2)	13.8 (9.4)
13.	Rothlind et al. (2007)	STN: 23 of 27 GPi: 19 of 28	33	9	STN: 61.4 (10.11) GPi: 60.2 (8.83)	STN: 15.2(3.21) GPi: 15.6(2.22)	STN: 12.9 (4.3) GPi: 13.3(6.4)	**15**	STN: 1925.9 (968.5) GPi: 1655.7 (874.4)	N.S	STN: 49.9(16.18) GPi: 44(15)	N.S
14.	Witjas et al. (2007)	40	30	10	59 (8)	N.S	12.4 (4.5)	**12**	1091.9 (374.8)	460.2 (299.1)	11.8 (5.8)	6.9 (14.5)
15.	Fraraccio et al. (2008)	15 of 16	9	6	58.1 (7.46)	11.3 (3.97)	13.6 (4.39)	**13.6** (4.39)	N.S	854.7 (500.03)	27.2 (10.2)	8.4 (5.1)
16.	Heo et al. (2008)	46	18	28	46.11 (10.6)	7.98 (4.48)	11.63 (5.67)	6 & **12**	798.94 (384.98)	N.S	20.28 (12.07)	N.S
17.	Zangaglia et al. (2009)	ODT 33 DBS 32	ODT 20 DBS 18	ODT 13 DBS 14	ODT 62.52 (6.8) DBS 58.8 (7.7)	ODT 7.5 (3.5) DBS 7.31 (3.21)	ODT 9.97 (4.8) DBS 11.8 (5)	**36**	617.19 (303.57)	722.66 (350.37)	ODT 19.16 (8.0) DBS 18.03 (8.3)	ODT 23.7 (8.9) DBS 22.81 (8.9)
18.	Castelli et al. (2010)	ODT 31 DBS 27	ODT 17 DBS 16	ODT 16 DBS 15	ODT 60.6 (6.7) DBS 60.2 (6.6)	ODT 8. (4.1) DBS 9.0 (4.1)	ODT 15.3 (5.1) DBS 15.6 (5.2)	**12**	DBS 1046.1 (436.4) ODT 1071.3 (370.3)	N.S	ODT 49.6 (11.9) DBS 55.0 (11.3)	N.S
19.	Follett et al. (2010)	STN: 147 GPi: 152	GPi 133 STN 116	GPi 19 STN 31	GPi: 61.9 ± 8.7 STN: 61.8 ± 8.7	N.S	GPi: 11.5 ± 5.4 STN: 11.1 ± 5.0	**24**	GPi: 1361 ± 545 STN: 1295 ± 585	GPi: 1118 ± 562 STN: 887 ± 545	GPi: 41.8 ± 13.1 STN: 43.0 ± 15.0	GPi: N.S STN: N.S
20.	Kishore et al. (2010)	45	27	18	55.4 ± 10.9	N.S	11.1 ± 5.7	**12,** 36, 60	669.8 ± 359.7	415.3 ± 365.6	48.1 ± 13.6	46.6 ± 18.6
21.	Mikos et al. (2010)	ODT 19 DBS 24	ODT 12 DBS 20	ODT 7 DBS 4	ODT 64.7 (6.6) DBS 61.7 (4.9)	ODT 15.4 (2.9) DBS 14.1 (2.6)	ODT 6.3 (5.7) DBS 11.5 (5.3)	**16**	1110.0 (533.5)	N.S	ODT 25.3 (8.5) DBS 22.9 (8.1)	N.S
22.	Smeding et al. (2011)	145	ODT: 22 STN: 63	ODT: 18 STN: 22	ODT: 63.5 (9.2) STN: 58.4 (7.8)	ODT: 12.2 (2.9) STN: 11.1 (2.9)	ODT: 10 (7.15) STN: 13 (10.18)	**12**	ODT: 545 (405.886) STN: 800 (553. 1174)	N.S	N.S	N.S
23.	Williams et al. (2011)	ODT: 18 STN: 19	ODT:15 STN:10	ODT: 3 STN: 9	ODT: 66.6 (9.0) STN: 62.1 (10.3)	ODT: 16.6 (1.20) STN: 13.6 (1.71)	ODT: 7.50 (4.22) STN: 10.1 (6.24)	**24**	ODT: 468.4 (293.0) STN: 1017.6 (411.2)	N.S	N.S	N.S
24.	Zibetti et al. (2011)	STN: 14	STN:9	STN: 5	STN: 60.4 (6.5)	N.S	STN: 17.0 (4.7)	**12** & 60	955 (406)	412 (265)	16.5 (11.0)	11.4 (6.8)
25.	Sjöberg et al. (2012)	B: 10 U: 6	B: 6 U: 5	B: 4 U: 1	B: 60.6 (6.8) U: 59.5 (11.1)	N.S	B: 8 (3.08) U: 5.1 (2.8)	6 & **18**	B: 428.1 (350) U: 435.3 (461.3)	N.S	N.S	N.S
26.	Yamanaka et al. (2012)	30	7	23	61.0 ± 9.1	12.5 ± 4.5	11.5 ± 5.7	1 & **12**	654 ± 273 (325–1475)	170 ± 151 (0–550)	22.4 ± 9.1 (1–42)	13.6 ± 6.9 (0–28)
27.	Kim et al. (2013)	36	18	18	56.8 ± 8.0	N.S	9.7 ± 4.1	6 & **1**2	1038.7 ± 473.9	266.0 ± 317.4	16.8 ± 10.0	15.8 ± 9.0
28.	Schuepbach et al. (2013)	ODT: 127 STN: 124	ODT: 85 STN: 94	ODT: 42 STN: 30	ODT: 52.2 ± 6.1 STN: 52.9 ± 6.6	N.S	ODT: 7.7 ± 2.7 STN: 7.3 ± 3.1	5. **12**. 24	ODT: 950.3 ± 21.3 STN: 935.6 ± 21.5	ODT: 1196.1 ± 18.8 STN: 572.3 ± 19.4	ODT: 950.3 ± 21.3 STN: 935.6 ± 21.5	ODT: 1196.1 ± 18.8 STN: 572.3 ± 19.4
29.	Asahi et al. (2014)	11	6	5	60.5 (43—72)	10.6	10.1 (5–14)	3. **12**. 60. 120	487.6 ± 150.5	296.7 ± 176.3	18.5 ± 9.1	13.5 ± 5.0
30.	Janssen et al. (2014)	26	18	8	58.0 ± 6.9 (43 − 70)	N.S	12.7 ± 5.1 (1 − 20)	3. **12**. 60. 120	824 ± 479	490 ± 298	21.2 ± 12.7	13.0 ± 6.4
31.	Merola et al. (2014)	134	78	56	60.32 ± 6.12 (40–70)	N.S	13.93 ± 4.78 (7–28)	**12**. 36. 60	1.061.94 ± 357.04 (100–2.300)	N.S	1.68 ± 1.47	N.S
32.	Rizzone et al. (2014)	26 of 30	18	8	68.5 ± 7.2 (54—81)	N.S	15.3 ± 5.8 (7/28)	**12**. 60. 132	1058.2 ± 566.8	451.7 ± 345.3	20.7 ± 10.0 (5—45)	15.7 ± 9
33.	Jiang et al. (2015)	17 of 24	9	8	59.4 ± 9.3 64.2	N.S	9.3 ± 2.9	**12**. 36. 60	660.4 ± 210.1	361.3 ± 250.9	15.6 ± 6.2	13.8 ± 5.8
34.	Odekerken et al. (2015)	128 of 142 STN: 56 GPi: 58	STN: 42 GPi: 40	STN: 14 GPi: 18	STN: 60.3 (7.4) GPi: 59.2 (7.7)	STN: 12.4 (3.4) GPi: 11.5 (2.8)	STN: 12.3 (5.5) GPi: 10.9 (4.0)	**12**	STN: 1.200 (900–1428.8) GPi: 1.226 (892.5–1655	N.S	STN: 44·4 (15·5) GPi: 43·8 (13·5)	STN: 24·1 (14·4) GPi: 32·4 (12·6)
35.	Tang et al. (2015)	27	18	9	55.53 ± 6.10	10.72 ± 5.34	10.12 ± 3.82	6. **12**	N.S	N.S	14.28 ± 8.30 (3–31)	16.50 ± 9.28 (4–39)
36.	Tramontana et al. (2015)	STN: 15 ODT: 15	STN: 14 ODT: 13	STN: 1 ODT: 2	STN: 60 (6.8) ODT: 60 (7)	N.S	N.S	**12**. 24	N.S	N.S	STN: 11.1 (6.9) ODT: 12.3 (6.4)	N.S
37.	Boel et al. (2016)	STN: 62 of 63 GPi: 58 of 65	STN: 44 GPi: 44	STN: 26 GPi: 24	STN: 60.9 (7.6) GPi: 59.1 (7.8)	N.S	STN: 12 (5.3) GPi: 10.8 (4.2)	**12.** 36	STN: 43 (68) GPi: 43 (69)	N.S	STN: 44·4 (15·5) GPi: 43·8 (13·5)	STN: 24·1 (14·4) GPi: 32·4 (12·6)
38.	Tröster et al. (2017)	101	63	38	60.6 (8.3)	N.S	12.1 (4.9)	3 & **12**	N.S	N.S	N.S	N.S
39.	Foki et al. (2018)	STN: 19 ODT: 25	DBS: 7 ODT: 14	DBS: 12 ODT: 14	DBS: 59.5 (10.2) ODT: 63 (6.6)	DBS: 10.1 (2.3) ODT: 10.5 (2.3)	N.S	**12**	N.S	N.S	DBS: 25.4 ± 11.4 ODT: 28.4 ± 12.7	N.S
40.	Acera et al. (2019)	40 of 50	18	32	62.2 (8.2)	N.S	14.1 (6.3)	**12**. 60	887.44 (412.86)	661.97 (413.37)	17.42 (7.16)	16.76 (7.23)
41.	Liu et al. (2019)	45	21	24	61.80 ± 8.08	N.S	11.16 (5.2)	**12**	996.67 ± 398.72	417.85 ± 235.51	48.49 ± 13.08 19.98	31.60 ± 12.20
42.	Pusswald et al. (2019)	STN:22 ODT: 28	DBS: 10 ODT: 15	DBS: 12 ODT: 13	DBS: 59.69 (10.28) ODT: 63.1 (7.43)	DBS: 10.77 (3.69) ODT: 10.57 (2.64)	N.S	**12**	N.S	N.S	N.S	N.S
43.	Dietrich et al. (2020)	32	20	12	62.3	10.3 ± 1.7	11.0 ± 5.3	**24**	1263 ± 861	1020 ± 679	36 ± 18	14
44.	Jost et al. (2021)	STN: 40 ODT: 40	DBS: 25 ODT: 27	DBS: 15 ODT: 13	DBS: 62.2 (8.6) ODT: 63.8 (10.4)	N.S	DBS: 9.7 (4.7) ODT: 8.3 (4.9)	**36**	DBS: 1066 (468.2) ODT: 885.2 (355.3)	DBS: 664.7 (436.6) ODT: 961.3 (397.8)	N.S	N.S
45.	Leimbach et al. (2020)	STN: 19 ODT: 9	DBS: 12 ODT: 5	DBS: 7 ODT: 4	DBS: 57.42 (7.5) ODT: 59.33 (6.12)	DBS: 13.87 (3.49) ODT: 16.11 (4.43)	DBS: 13.47 (3.91) ODT: 10.11 (5.02)	**12**/24	N.S	N.S	DBS: 49.53 (17.91) ODT: 18.71 (12.48)	DBS: 21 (13.53)
46.	Mulders et al. (2021)	49	31	18	61.1 (8.5)	N.S	11.14 (2.64)	**12**	1346.1 (629.9)	617.9 (476.5)	37.7 (12.5)	18.8 (9.8)
47.	You et al. (2020)	STN:20 ODT: 20	DBS: 10 ODT: 10	DBS: 10 ODT: 10	DBS: 59 (4.23) ODT: 58.35 (6.77)	N.S	DBS: 9.55 (2.35) ODT: 8.65 (2.3)	**12**	DBS: 785 (236) ODT: 773 (352)	DBS: 407 (197) ODT: 832 (260)	DBS: 48.5 (14.4) ODT: 46.7 (11.1)	DBS: 45.8 (10.4) ODT: 47.3 (11.1)
48.	Volonté et al. (2021)	18	13	5	45 ± 7 (30–56)	10 ± 4 (3–16)	11 ± 4 (6–20)	**12,** 108, 168	1163.8 ± 375.3 (520.4–1790)	690.2 ± 426.5 (140.7–1737.5)	40.9 ± 11.2 (21–65)	35.7 ± 9.6 (18–59)

*LEDD* levodopa-equivalent daily dose, *STN* subthalamic nucleus, *GPi* internal globus pallidus, *N.S.* not specified, *DBS* deep brain stimulation, *U* unilateral electrical stimulation, *B* bilateral electrical stimulation, *ODT* optimal drug therapy (control group)

• time from the DBS (months): in bold the time point after DBS taken into consideration by our analysis

The standardized mean difference (SMD) computed as Hedges’ g and sampling variance for each included study were calculated using the Comprehensive Meta-Analysis Software, while summary analyses, the likelihood of publication bias and heterogeneity tests, were computed using the “metafor package” for R, version2.4–0 (Viechtbauer, 2010). Pre- versus post- SMDs have some limitations, namely in uncontrolled designs it is often impossible to disentangle which proportion of the SMD (in our case Hedges’ g) is due to the treatment and which to spontaneous recovery or other uncontrolled variables. In other words, the pre-post SMDs are not always informative about the effects of the treatment, and these types of studies often suffer high levels of heterogeneity. Another important issue with pre-post SMDs is that the scores on the outcome measures at pre-test and those at post-test are not independent of each other. To account for the correlation between these two scores, and because the value for this correlation is seldom reported, we assumed as fixed value the Rosenthal's conservative estimate of 0,7, as many previous studies did (Hofmann et al., 2014; Johnsen & Friborg, 2015). Considering that this value is not based on empirical data, we also computed the same analysis with other three correlation points (0,0, 0,5, and 0,9) to verify whether there was any change by modifying the correlation estimate.

When a control group was included, Hedges’ g and variance were calculated for each study based on the pre-post means and standard deviation, the number of participants from both control and DBS groups, and the estimated correlations of 0,0, 0,5, 0,7, and 0,9. We only report the results with a correlation of 0,7, since we observed that what was statistically significant remained so independent of the correlation point.

### Study Quality Assessment

The methodological quality of the included studies was assessed using the Physiotherapy Evidence Database (PEDro) tool and a grade for the level of evidence was assigned to each study according to the modified Sackett Scale (see Table 1 for the level of evidence; Sackett et al., 2000; Moseley et al., 2002). PEDro consists of a checklist of 11 yes-or-no questions (Table S1 in Appendix A, Supplementary data) assessing the quality of clinical trials. The PEDro scale is considered a valid and comprehensive instrument previously applied in systematic reviews (de Morton, 2009; McIntyre et al., 2016). Items can be scored as either present (1) or absent (0), and the total score is obtained by summation. Higher values indicate greater quality (9–10, excellent; 6–8, very good; 4–5, good; < 4, poor; Foley et al., 2003). Criterion 1, which relates to external validity, is not used to calculate the PEDro score.

The Sackett Scale includes five levels of evidence. Level 1 refers to meta-analysis and “high-quality” RCTs (PEDro score ≥ 6). Level 2 evidence is also derived from RCTs but from those with PEDro scores less than six, while Level 3 evidence refers to case‐control studies. Levels 4 and 5 comprise uncontrolled pre-and post-treatment tests, observational studies, case studies, or single-subject series with no multiple baselines. Overall evidence was qualified using the grading of recommendations, assessment, development, and evaluations (GRADEpro GDT, https://gradepro.org) and the Meader et al. (2014) GRADE assessment checklist (Table S2, in Appendix A. Supplementary data). GRADE provides a transparent approach and guidance on rating the overall quality of research evidence indicating four levels of evidence along a continuum (i.e., high, moderate, low, and very low) based on five factors including 1) risk of bias, 2) inconsistency, 3) indirectness, 4) imprecision, and 5) publication bias (Meader et al., 2014).

For each meta-analysis, the pooled effect, and the level of heterogeneity, by means of the Q and I^2^ statistics were calculated (Higgins & Thompson, 2002). The Q-statistic, representing the ratio of observed variation to within-study variance, indicates how much of the overall heterogeneity can be attributed to between-studies variation. Being a null hypothesis significance test, it is assessing the null hypothesis that all studies are examining the same effect. Therefore, when statistically significant, it implies that the included studies do not share a common effect size (Higgins & Thompson, 2002; Quintana, 2015). I^2^ (i.e., total heterogeneity or total variability) is a percentage which estimates the proportion of the observed variance reflecting a real difference in effect sizes, or the actual difference between studies (Borenstein et al., 2017). I^2^ values of 25%, 50%, and 75%, represent low, moderate, or high inconsistency, respectively. Influential cases were identified using the “inf” function from the “metafor package” for R (Del Re, 2015; Kovalchik, 2013; Polanin et al., 2016). To identify studies that have may disproportionately contributed to heterogeneity and the overall result, we used (Baujat et al., 2002) plot. The horizontal axis illustrates study heterogeneity and the vertical axis illustrates the influence of a study on the overall result. We also applied a set of diagnostics derived from standard linear regression, available within the “metafor package”, in order to spot potential outliers which could influence the observed heterogeneity (Viechtbauer & Cheung, 2010).

Meta-analysis publication bias may be due to various elements, such as the fact that we explicitly included only peer-reviewed, English-written papers, or that experiments with small effect sizes are more likely to remain unpublished. The likelihood of publication bias was assessed graphically by using the funnel plot tool together with the Egger’s regression test (Egger et al., 1997) and the rank correlation test (Begg & Mazumdar, 1994). The trim and fill method, which imputes “missing” studies to create a more symmetrical funnel plot, (Duval & Tweedie, 2000) was used for bias correction only if the previously mentioned tests were significant, since a p-value < 0.05 is consistent with a non-symmetrical funnel plot.

To avoid a large number of figures and tables, some of the materials, such as the risk of bias evaluation tables and the Baujat and Funnel plots, are found in Appendix A in the Supplementary Data.

## Results

**Fig. 1:**
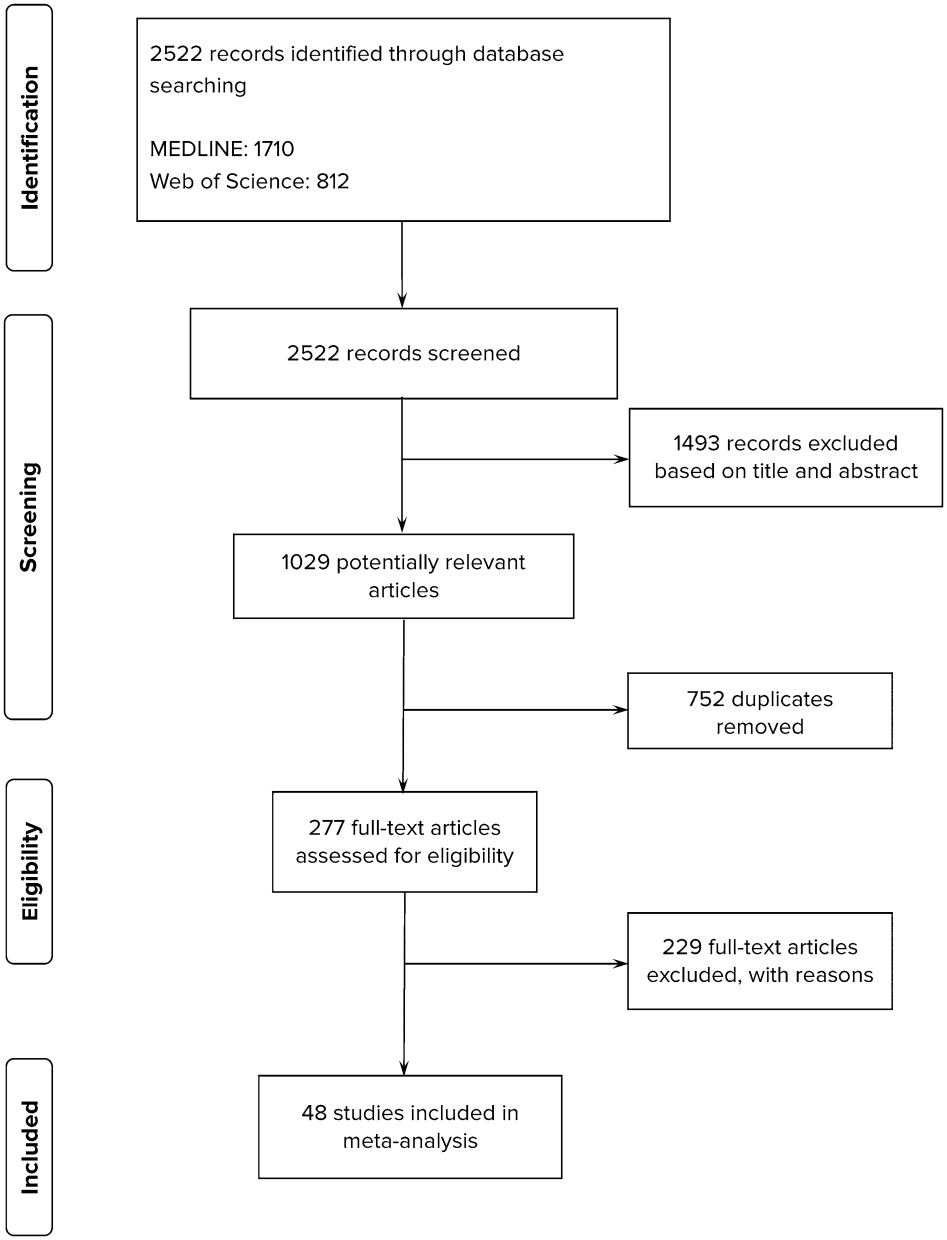
Flow Diagram of study selection and inclusion

We retrieved 2522 citations. Duplicates and studies that did not satisfy the inclusion criteria as revealed by the title or abstract were excluded, and 277 papers underwent full review (Fig. 1.), resulting in 48 accepted articles (Table 1).

### Studies Characteristics

Thirty-four studies were within-subjects designs (uncontrolled pre-post DBS) and fourteen papers had a PD (no DBS) control group. With the exception of two RCTs (Schuepbach et al., 2013; Tramontana et al., 2015), no studies were blinded or random. The methodological quality of the RCTs was high, level 1b evidence. The 14 studies with a control group were rated as level 3 on the modified Sackett Scale (McIntyre et al., 2016), while the uncontrolled pre-post studies were considered as level 4 evidence (Tables 1 and A.3). The main risk of bias was due to the methodological limitations of the open-label design. Cognitive decline may occur in patients with PD over time, and in serial (test–retest) neuropsychological assessments, a repeated performance can improve due to practice effects when no parallel versions are used. Another main risk of bias was the lack of randomization and allocation concealment in between-subjects design.

**Table 3:** Summary of all the meta-analysis results, grouped by domains

**Domain & Comparison**	**Excluded Studies**	**Number of Studies**	**Effect Size Summary**	**95% CI**		**Z value**	**p-value for Z**	**Q—Test for Heterogeneity**	**p- value for Q**	**τ**^**2**^** estimated amount of total heterogeneity**	**Influence Test**	**Regression Test for Funnel Plot Asymmetry**	**Rank Correlation Test for Funnel Plot Asymmetry (Kendall's tau)**
**Memory**													
Delayed recall		24	-0,13 *	-0,23	-0,02	-2,41	0,02*	56,3	0,00	0,04 (SE = 0,02)	none	z = -0,09, p = 0,93	tau = -0,01, p = 0,98
Delayed recall: DBS vs. ODT		5	-0,40 *	-0,75	-0,05	-2,23	0,02 *	8,85	0,06	0,08 (SE = 0,11)	(Zangaglia et al., 2009; You et al., 2020)	z = 1,13, p = 0,26	tau = 0,40, p = 0,48
Backward Digit Span		9	0,11	-0,02	0,23	1,70	0,09	6,07	0,64	0 (SE = 0,01)	Yamanaka et al. (2012)	z = 0,88, p = 0,37	tau = 0,22, p = 0,48
Immediate Recall		16	-0,06	-0,21	0,09	-0,74	0,45	45,42	0,00	0,06 (SE = 0,03)	none	z = -0,62, p = 0,53	Tau = 0,0, p = 1,0
**Executive Function and Attention**													
Phonemic fluency STN		31	-0,42*	-0,51	-0,33	-8,96	< 0,0001 *	83,08	0,00	0,0323 (SE = 0,01)	Klempírová et al. (2007)	z = -2,76, p = 0,01	tau = -0,15, p = 0,24
Phonemic fluency STN	Moretti et al. (2003)	30	-0,40 *	-0,49	-0,32	-9,21	< 0,0001 *	63,29	0,00	0,0258 (SE = 0,01)	none	z = -1,19, p = 0,24	tau = -0,09, p = 0,48
Phonemic fluency GPi		6	-0,30*	-0,55	-0,04	-2,30	0,02 *	16,03	0,01	0,0719 (SE = 0,06)	Pillon et al. (2000)	z = 1,49, p = 0,14	tau = 0,47, p = 0,27
Phonemic fluency: DBS vs. ODT		8	-0,56 *	-0,79	-0,33	-4.83	< 0,0001 *	8,64	0,28	0,0230 (SE = 0,05)	none	z = 1,15, p = 0,26	tau = 0,28, p = 0,40
Stroop Color and Word STN		21	-0,30 *	-0,39	-0,22	-6,9	< 0,0001 *	32,87	0,03	0,0144 (SE = 0,01)	none	z = -2,3, p = 0,02	tau = -0,12, p = 0,49
*Publication bias assessed by trim and fill*		26	-0,26 *	-0,34	-0,18	-6,32	< 0,0001 *	42,13	0,02	0,0149 (SE = 0,01)	Estimated number of missing studies on the right side: 5 (SE = 3.05)		
Stroop Color and Word GPi		6	-0,16	-0,38	0,05	-1,49	0,13	17,18	0,00	0,0444 (SE = 0,04)	none	z = 0,42, p = 0,68	tau = 0,2, p = 0,72
Stroop Color and Word: DBS vs. ODT		5	-0,45 *	-0,74	-0,15	-2,97	0,00 *	6,84	0,14	0,0317 (SE = 0,07)	none	z = -0,59, p = 0,54	tau = -0,20, p = 0,82
**Language**													
Semantic fluency STN		28	-0,48*	-0,55	-0,41	-12,9	< 0,0001 *	46,6	0,01	0,0137 (SE = 0,01)	none	z = -1,23, p = 0,21	tau = -0,05, p = 0,68
Semantic fluency GPi		6	-0,50*	-0,59	-0,40	-9,94	< 0,0001 *	6,82	0,23	0,0000 (SE = 0,01)	Follett et al. (2010)	z = 0,21, p = 0,83	tau = -0,07, p = 1,00
Semantic fluency: DBS vs. ODT		7	-0,49 *	-0,70	-0,27	-4,35	< 0,0001 *	3,16	0,79	0,0000 (SE = 0,05)	none	z = 0,45, p = 0,65	tau = 0,24, p = 0,56
**General emotional state**													
Depression STN		27	0,34 *	0,04	0,65	2,22	0,02*	462,1	0,00	0,6172 (SE = 0,18)	Schuepbach et al. (2013)	z = 0,69, p = 0,48	tau = -0,02, p = 0,93
Depression STN	Schuepbach et al., 2013)	26	0,21*	0,07	0,34	3,04	0,002*	153,7	0,00	0,0936 (SE = 0,04)	none	z = -0,1, p = 0,9	tau = -0,08, p = 0,57
Depression GPi		4	0,11 *	0,01	0,21	2,22	0,03 *	4,79	0,19	0,0000 (SE = 0,01)	(Follett et al., 2010; Boel et al., 2016)	z = 2,04, p = 0,04	tau = 0,66, p = 0,33
*Publication bias assessed by trim and fill*		5	0,10 *	-0,00	0,19	1,96	0,050*	7,18	0,13	0,0000 (SE = 0,01)	Estimated number of missing studies on the left side: 1 (SE = 1,5779)		
Anxiety STN		10	0,30 *	0,10	0,50	3,00	0,01 *	30,04	0,00	0,0691 (SE = 0,05)	Tang et al. (2015)	z = 0,87, p = 0,38	tau = 0,20, p = 0,48

When the publication bias tests revealed significant asymmetry the trim and fill assessment results were reported immediately below the original analysis

Articles were excluded when they were identified as outliers, but for transparency, we present all the results (with and without the excluded papers)

^*^ indicates the statistical significand SMDs

### Participants’ Characteristics and Intervention

This review includes 2039 adults with a clinical diagnosis of PD undergoing DBS surgery and 271 PD control participants (ODT [optimal drug therapy]). 1768 patients received STN stimulation, and 271 received GPi stimulation. Only 36 patients received unilateral DBS. In the DBS participants, LEDD was lowered in almost all cases (Table 2).

The participants’ characteristics were heterogeneous among studies especially concerning the time interval from PD diagnosis, age, and inclusion criteria (Table 2).

Regarding the DBS intervention, unilateral or bilateral STN was targeted in all the included studies, but only seven studies targeted STN and GPi separately. The stimulation parameters were also heterogeneous and not always specified. For this reason, we could not use this type of data as covariates in our analysis. For example, different types of electrodes from monopolar (Mikos et al., 2010) to tetrapolar (Acera et al., 2019) were used, pulse width ranged between 94,0 ± 10,56 μs (Fraraccio et al., 2008) and 60,5 ± 10,9 μs (Pillon et al., 2000), and usually a high-frequency stimulation, for example, 183,5 Hz (Rothlind et al., 2007) or 130 – 135 Hz (Asahi et al., 2014), was applied, while voltage varied between 2 and 4 V (Boel et al., 2016; Fraraccio et al., 2008; Odekerken et al., 2015; Pillon et al., 2000).

## Meta-analysis Results

As previously mentioned, we took into consideration only specific neuropsychological tests that cover four cognitive areas. After inspecting the literature, we kept those tests that were more frequently used in order to have enough analysis power. When it occurred that the instruments were so heterogeneous that it was not possible to choose one test, but we had evaluated the specific domain as important, we combined different neuropsychological tests (this is true for delayed recall, immediate recall, depression and anxiety).

The standardized mean differences (SMD) were pooled together using the random-effects model regardless of the heterogeneity of test results (Q or I2) since there was a certain amount of variance between studies due to their characteristics (e.g., stimulation parameters, stimulation areas, patients’ characteristics). All the meta-analytic results are listed in Table 2.

### DBS Effects on Memory

#### Delayed Recall

Twenty-four papers were considered and nine different assessment instruments were used, including (a) *Rey Auditory Verbal Learning Test – delayed recall* (Mulders et al., 2021; Acera et al., 2019; Boel et al., 2016; Odekerken et al., 2015; Heo et al., 2008; Rizzone et al., 2014; Smeding et al., 2011; Williams et al., 2011), (b) *Grober and Buschke Verbal Learning Test – delayed free recall* (Dujardin et al., 2001; Funkiewiez, 2004; Pillon et al., 2000), (c) *Hopkins Verbal Learning Test-Revised – delayed recall* (Follett et al., 2010; Mikos et al., 2010) (d) *Wechsler Memory Scale – delayed logical memory* (Fraraccio et al., 2008; Klempírová et al., 2007; You et al., 2020; Zangaglia et al., 2009), (e) *California Verbal Learning Test – long delay free recall* (Janssen et al., 2014; Woods et al., 2001), (f) *Brief Visuospatial Memory Test–Revised* (Rothlind et al., 2007), (g) *Repeatable battery for the assessment of neuropsychological status – delayed memory index* (Asahi et al., 2014), (h) *Chinese Auditory Verbal Learning Test – delayed recall* (Tang et al., 2015) delayed recall, and (i) *Story recall test – delay free recall* (Volonté et al., 2021).

**Fig. 2:**
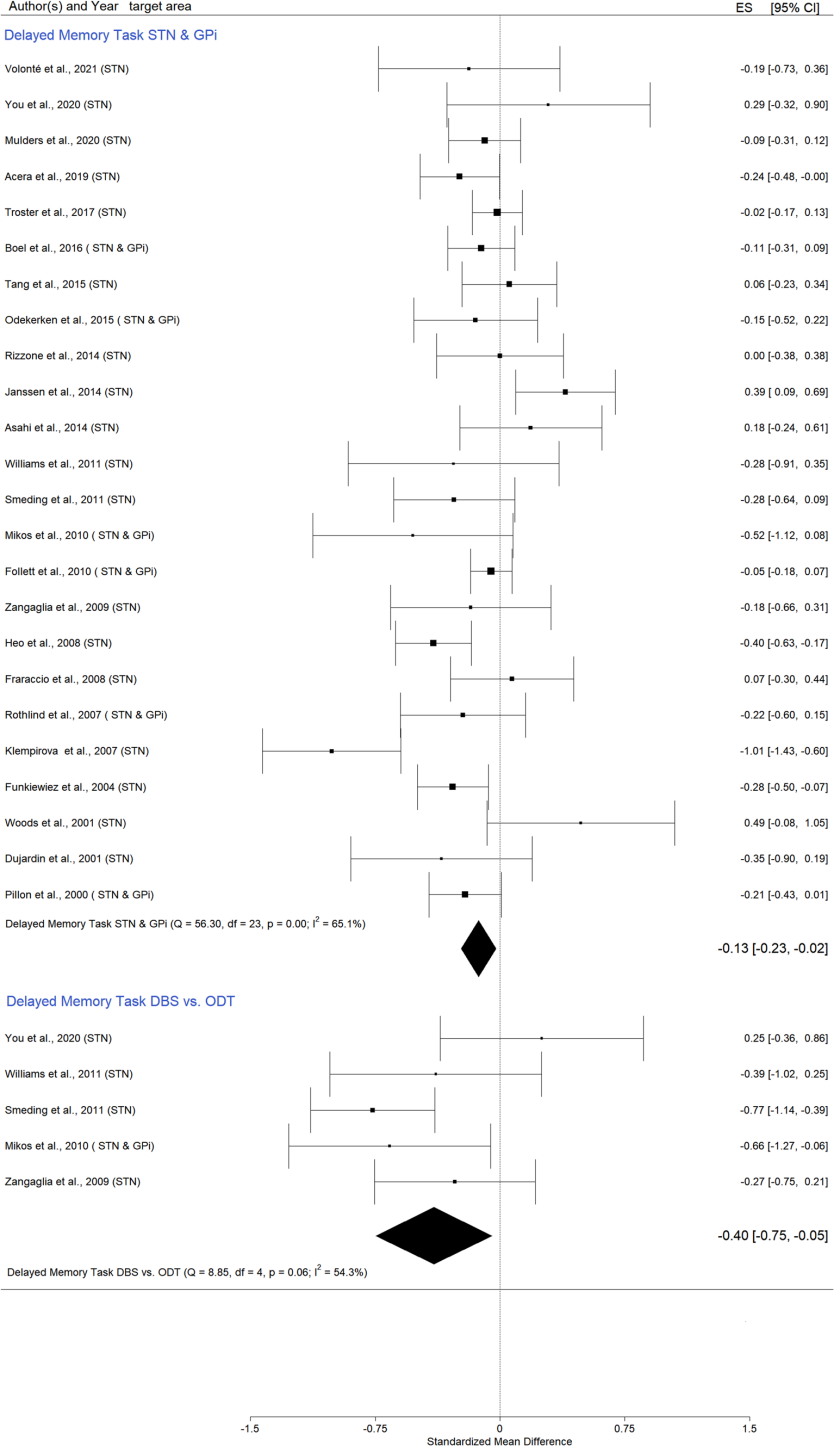
Forest plot—DBS effects on delayed recall

The *SMD for STN and Gpi combined* was small but statistically significant (Fig. 2, and in Appendix A – Figs. S1 and S2), Hedges’ g = -0,13 (95% CI = [-0,23; 0,02]; p = 0,02; K = 24, N = 1429).

Only studies including a control group, *DBS vs. ODT PD*, were analyzed separately in order to exclude possible confounding factors that characterize pre-post data. A statistically significant negative effect was observed in that the DBS PD patients’ scores were lower than those of the ODT PD group (Fig. 2, and in Appendix A Figs. S3 and S4), Hedges’ g = -0,40 (95% CI = [-0,75; 0,05]; p = 0,02; K = 5, N_control = 130, N_DBS = 200). We did not observe a funnel plot asymmetry, but two studies (You et al., 2020; Zangaglia et al., 2009) had a higher impact on the result.

#### Backward Digit Span

**Fig. 3:**
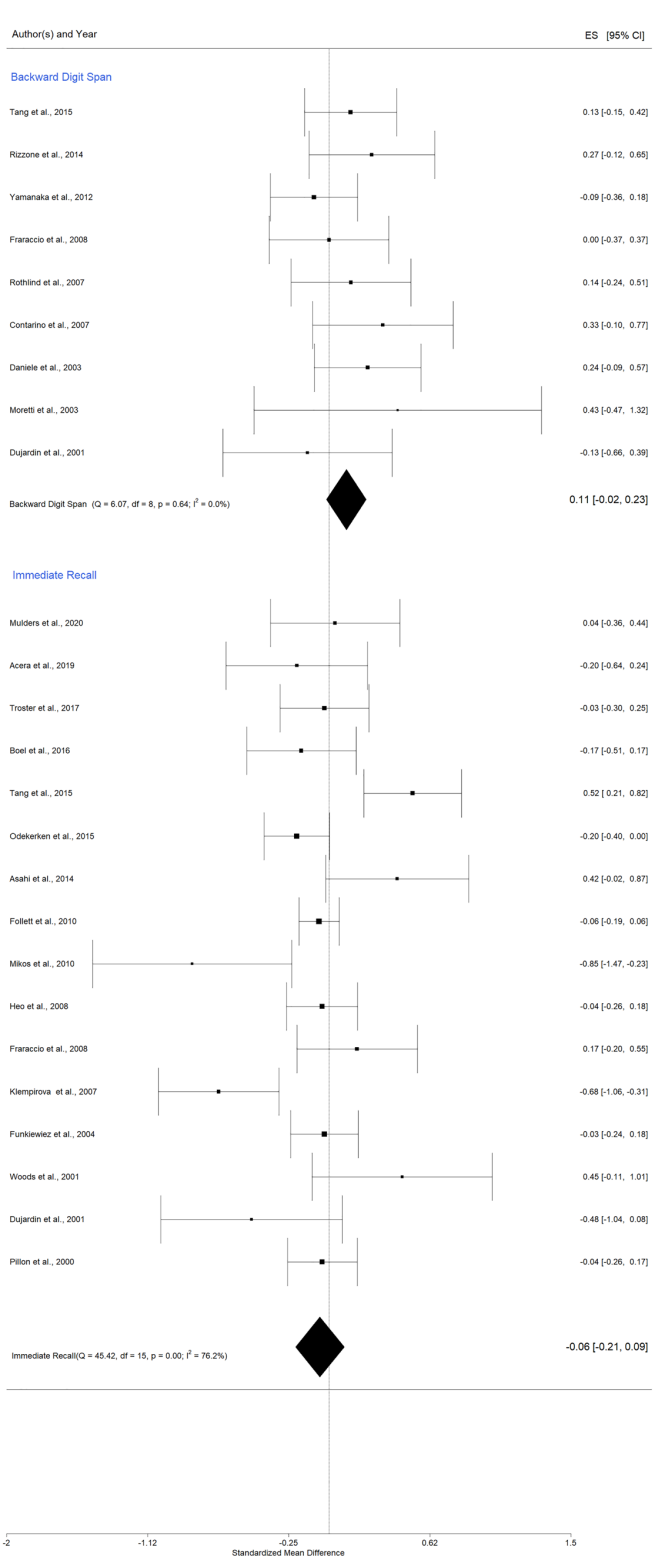
Forest plot - DBS effects on working memory and immediate recall

Analysis included nine studies (Contarino et al., 2007; Daniele et al., 2003; Dujardin et al., 2001; Fraraccio et al., 2008; Moretti et al., 2003; Rizzone et al., 2014; Rothlind et al., 2007; Tang et al., 2015; Yamanaka et al., 2012). Pooled data did not provide evidence of significant changes after DBS (Fig. 3), Hedges' g = 0,11 (95% CI = [-0,02; 0,23]; p = 0,09; K = 9, N = 156). The levels of heterogeneity among studies were low: Q test = 6.07, p = 0,64, and I^2^ was 0,00%, but we applied the random-effects model because the differences between studies in terms of participants’ characteristics were relevant. The Yamanaka et al. (2012) study was identified as an outlier by the Baujat plot (Fig. S5 in Appendix A). Publication bias was evaluated using the funnel plot, the Egger's regression intercept test, and the rank correlation test for funnel plot asymmetry (Kendall's tau). No evidence of publication bias was found (Fig. S6 in Appendix A, Table 3).

#### Immediate Recall

Analysis included 16 papers and nine different assessment instruments, including (a) *Grober and Buschke Verbal Learning Test – free immediate recall* (Dujardin et al., 2001; Funkiewiez, 2004; Pillon et al., 2000), (b) *Wechsler Memory Scale—logical immediate memory* (Fraraccio et al., 2008; Klempírová et al., 2007; Tröster et al., 2017), (c) *Hopkins Verbal Learning Test-Revised—logical memory* (Follett et al., 2010; Mikos et al., 2010), (d) *California Verbal Learning Test—immediate verbal list learning* (Woods et al., 2001), (e) *Rey–Kim Memory Battery – verbal memory immediate recall* (Heo et al., 2008), (f) *Repeatable battery for the assessment of neuropsychological status* (Asahi et al., 2014), (g) *Rivermead Behavioral Memory Test* (Odekerken et al., 2015), (h) *Chinese auditory verbal learning test*—*verbal memory* (Tang et al., 2015*), and (i) Rey Auditory Verbal Learning Test* (Boel et al., 2016; Acera et al., 2019; Mulders et al., 2021).

The random-effects meta-analysis, for STN and GPi combined, yielded a statistically non-significant result, with an overall effect size of Hedges' g = -0,06 (95% CI = [-0,21, 0,09]; p = 0,645; K = 16, N = 720), Fig. 3. We further used the Baujat plot to explore heterogeneity (Fig. S7 in Appendix A) and the funnel plot to assess publication bias (Fig. S8 in Appendix A). In the absence of publication bias, studies should be distributed symmetrically with larger studies appearing toward the top of the graph and clustered around the mean effect size and smaller studies toward the bottom. Data showed no potential outliers, and tests for publication bias indicated no need for bias correction, given that neither the rank correlation nor Egger's regression test was statistically significant.

### DBS Effects on Executive Function

#### Phonemic Verbal Fluency

**Fig. 4:**
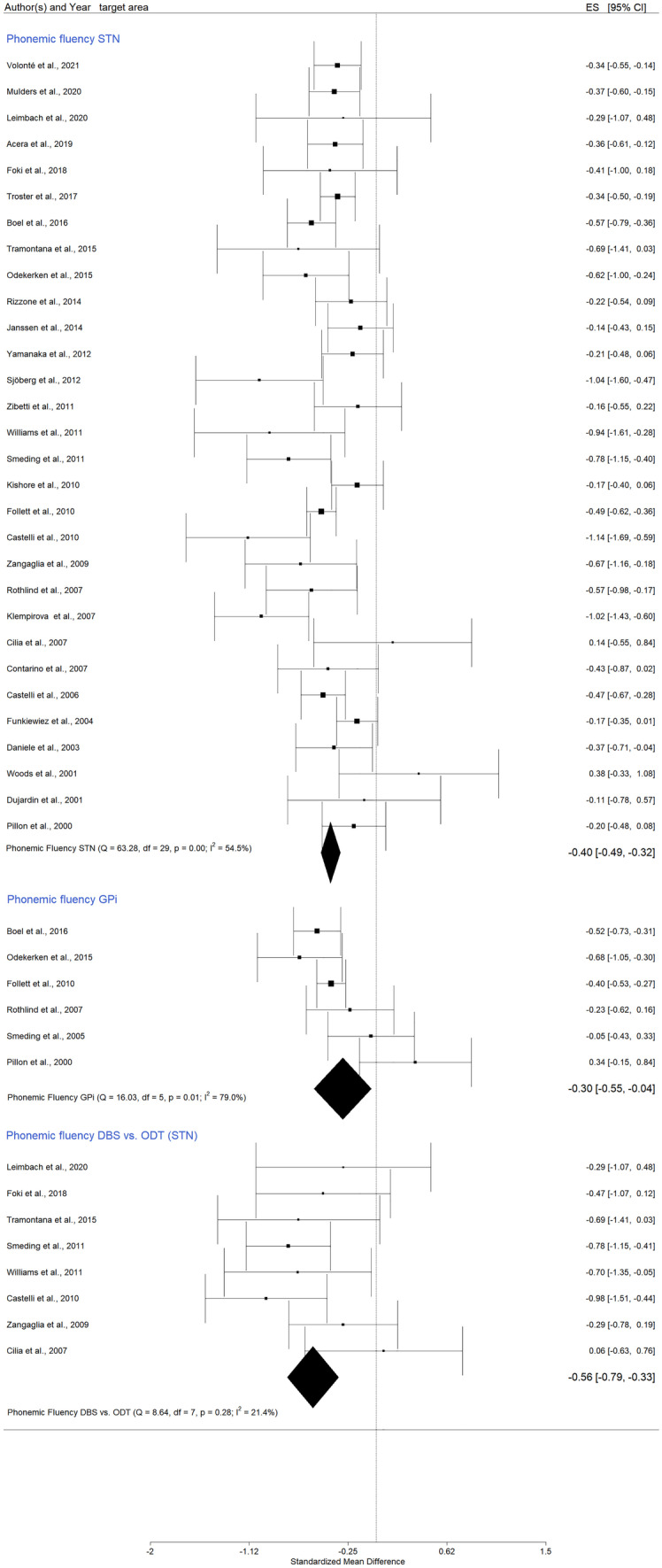
Forest plot – DBS effects on phonemic verbal fluency

Thirty-one studies investigated phonemic fluency *post STN DBS*. Hedges’ g value was -0,42 (95% CI = [-0,51; -0,33]; p < 0,0001; K = 31, N = 1326). In Appendix A, Fig. S9 shows the forest plot, while Figs. S10 and S11 indicate the Baujat plot and the Funnel plot respectively. Moretti et al. study (Moretti et al., 2003) was identified as a potential outlier. After its exclusion, the effect size did not significantly change, indicating a decrease in the phonemic fluency performance after DBS, Hedges’ g = -0,40 (95% CI = [-0,49; -0,32]; p < 0,0001; K = 30, N = 1308), (Fig. 4).

When only the *GPi stimulation studies* were pooled into the analysis, Hedges’ g had a value of -0.30 (95% CI = [-0,55; 0,04]; p = 0,02; K = 6, N = 304), (Fig. 4, and in Appendix A Figs. S13 and S14). This result was statistically significant, suggesting relevant differences between the pre-post phonemic fluency after GPi DBS. The heterogeneity was high (I^2^ = 79%) and the Baujat plot indicated Pillon et al. (2000) as a potential outlier (Pillon et al., 2000), (Fig. S13 in Appendix A). The funnel plot revealed no publication bias (Fig. S14 in Appendix A).

Eight studies compared *DBS and ODT PD* participants, and the results were statistically significant, characterized by low heterogeneity and no publication bias (Fig. 4, and in Appendix A Figs. S15 and S16), Hedges’ g = -0,56 (95% CI = [-0,79; -0,33]; p < 0.0001; K = 8, N_control = 183, N_DBS = 256).

#### Color–word Stroop Test

**Fig. 5:**
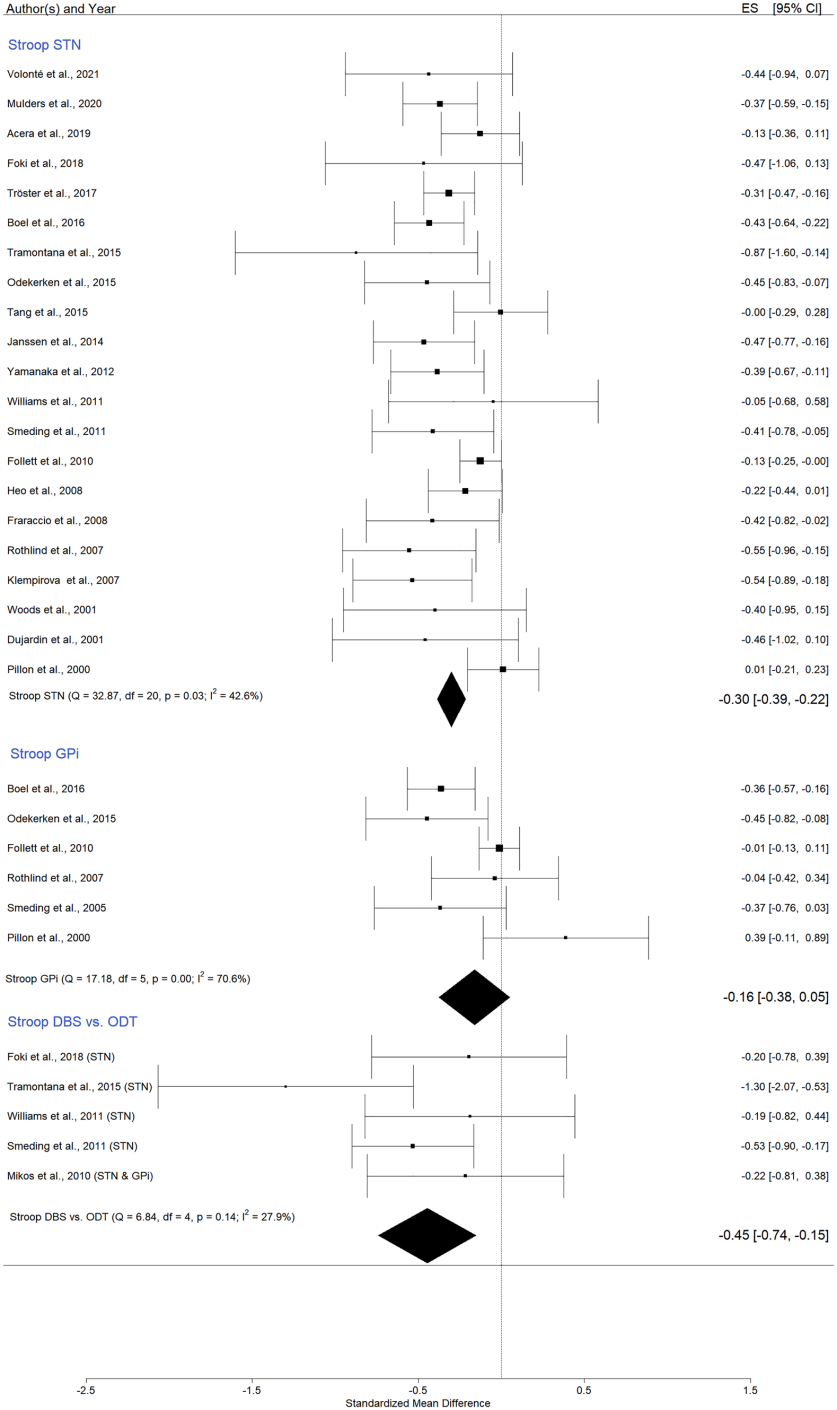
Forest plot – DBS effects on Stroop test (color–word)

We pooled 21 studies into the meta-analysis, and the result for *all STN* studies was statistically significant, Hedges’ g = -0,30 (95% CI = [-0,39; -0,22]; p < 0,0001; K = 21, N = 958, I^2^ = 42.6%), (Fig. 5, and in Appendix A Figs. S17 and S18). Because the funnel plot and regression test for asymmetry suggested a risk of publication bias (Appendix A Fig. S18), the trim and fill method was applied, estimating five missing studies on the right side, Hedges’ g = -0,26 (95% CI = [-0,34; -0,18]; p < 0,0001; K = 26). Data indicate that after STN DBS, the performance decreased.

Considering *only GPi stimulation*, the Hedges’ g had a value of =—0,16 (95% CI = [-0,38; 0,05]; p = 0,13; K = 6, N = 304, I^2^ = 70,6%), a small effect size that did not reach statistical significance (Fig. 5, and in Appendix A Figs. S19 and S20).

Comparing *pre-post DBS and ODT PD* patients an effect size of -0,45 was observed (95% CI = [-0,74; -0,15]; p = 0,003; K = 5, N_controls = 117, N_DBS = 182; I^2^ = 27.9%), a medium value indicating that after surgery the DBS group had statistically significant lower scores (Fig. S17, and in Appendix A Figs. S21 and S22).

### DBS Effects on Language

#### Semantic Fluency

Twenty-eight studies investigated STN DBS effects on semantic fluency. Six papers examined the GPi stimulation, and eight publications included a control group.

**Fig. 6:**
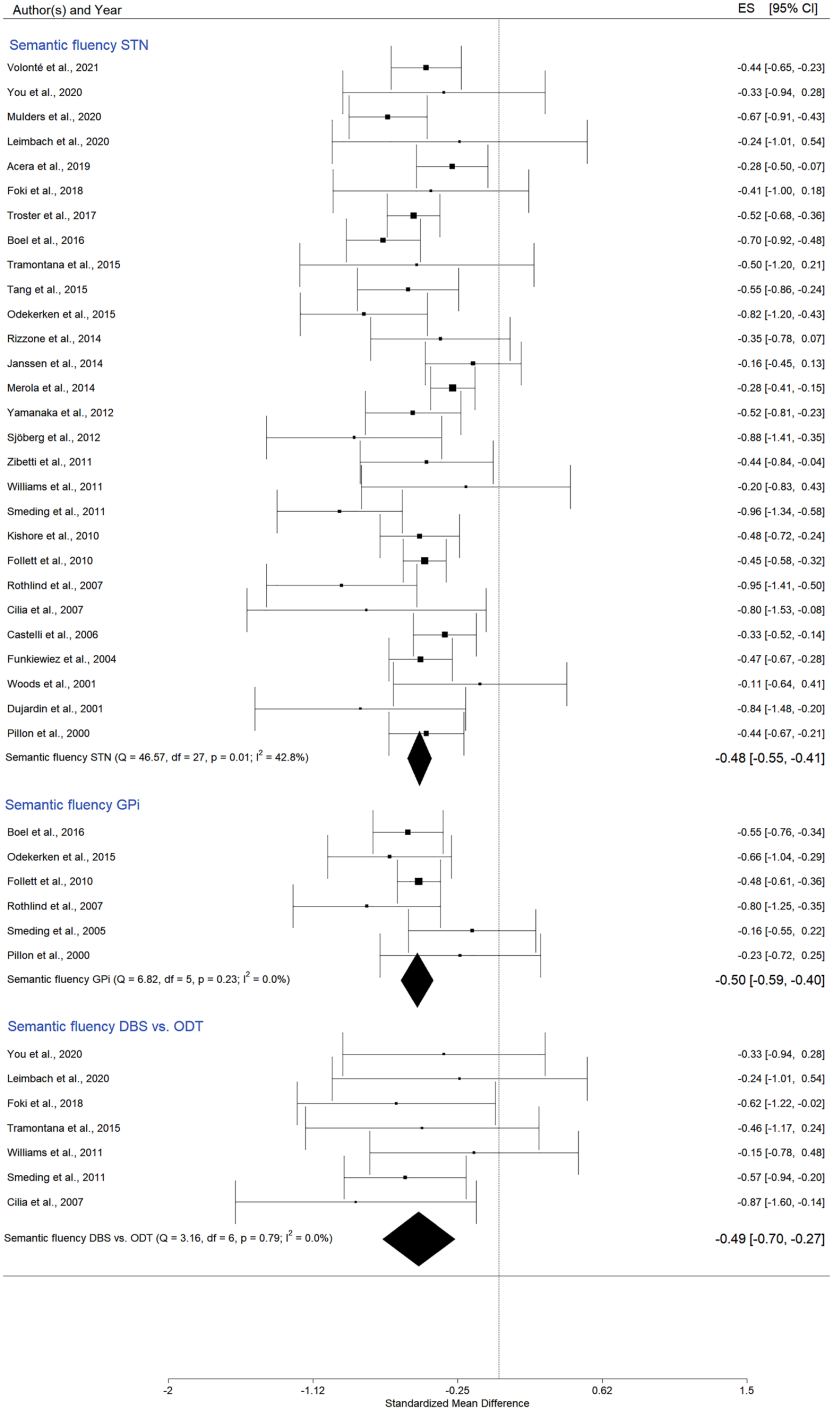
Forest plot – DBS effects on semantic verbal fluency

In the case of *STN stimulation*, SMD was -0,48 (95% CI = [-0,55; -0,41]; p < 0,0001; K = 28, N = 1378), (Fig. 6, and in Appendix A Figs. S23 and S24). The I^2^ of 42.8% indicated a moderate heterogeneity of the effect size. The pooled analysis also revealed statistically significant SMD for *GPi DBS*, Hedges’ g = -0,50 (95% CI = [-0,59; -0,40]; p = < 0,0001; K = 6, N = 304), (Fig. 6, and in Appendix A Figs. S25 and S26).

An additional subgroup meta-analysis on semantic fluency exploring the differences between *DBS and no-DBS PD* patients showed a Hedges’ g value of -0,49 (95% CI = [-0,70; -0,27]; p < 0,0001; K = 7, N_control = 139, N_DBS = 217), (Fig. 6, and in Appendix A Figs. S27 and S29). DBS patients obtained lower scores compared to no-DBS PD participants.

### DBS Follow-up Effects on Emotional State: Depression and Anxiety

All of the average scores of the psychometric scales, in which higher total scores indicate more severe symptoms (e.g., depression or anxiety), were multiplied by -1 to ensure that all scales pointed in the same direction. Specifically, an improvement of the investigated function will be located on the right part of the forest plot with a positive sign. In contrast, a lower score will be located on the left part of the forest plot, having a negative sign, and will indicate a decline of the cognitive function.

#### Depression 

Assessment was relatively consistent across the 27 included studies. The neuropsychological tests used for the evaluation follow. Seventeen used the *Beck Depression Inventory (BDI)* (Castelli et al., 2006; Dietrich et al., 2020; Follett et al., 2010; Funkiewiez, 2004; Heo et al., 2008; Janssen et al., 2014; Kim et al., 2013; Kishore et al., 2010; Liu et al., 2019; Pillon et al., 2000; Pusswald et al., 2019; Rothlind et al., 2007; Tang et al., 2015; Volonté et al., 2021; Witjas et al., 2007; Woods et al., 2001; Zibetti et al., 2011), four papers applied the *Montgomery–Åsberg Depression Rating Scale (MADRS)* (Acera et al., 2019; Ory-Magne et al., 2007; Schuepbach et al., 2013; Smeding et al., 2011), *three used the Hamilton Depression Rating Scale (HAM-D)* (Boel et al., 2016; Jiang et al., 2015), two publications applied the *Zung Self-Rating Depression Scale* (Daniele et al., 2003; Rizzone et al., 2014), and one the *Hospital Anxiety and Depression Scale* (Jost et al., 2021).

**Fig. 7:**
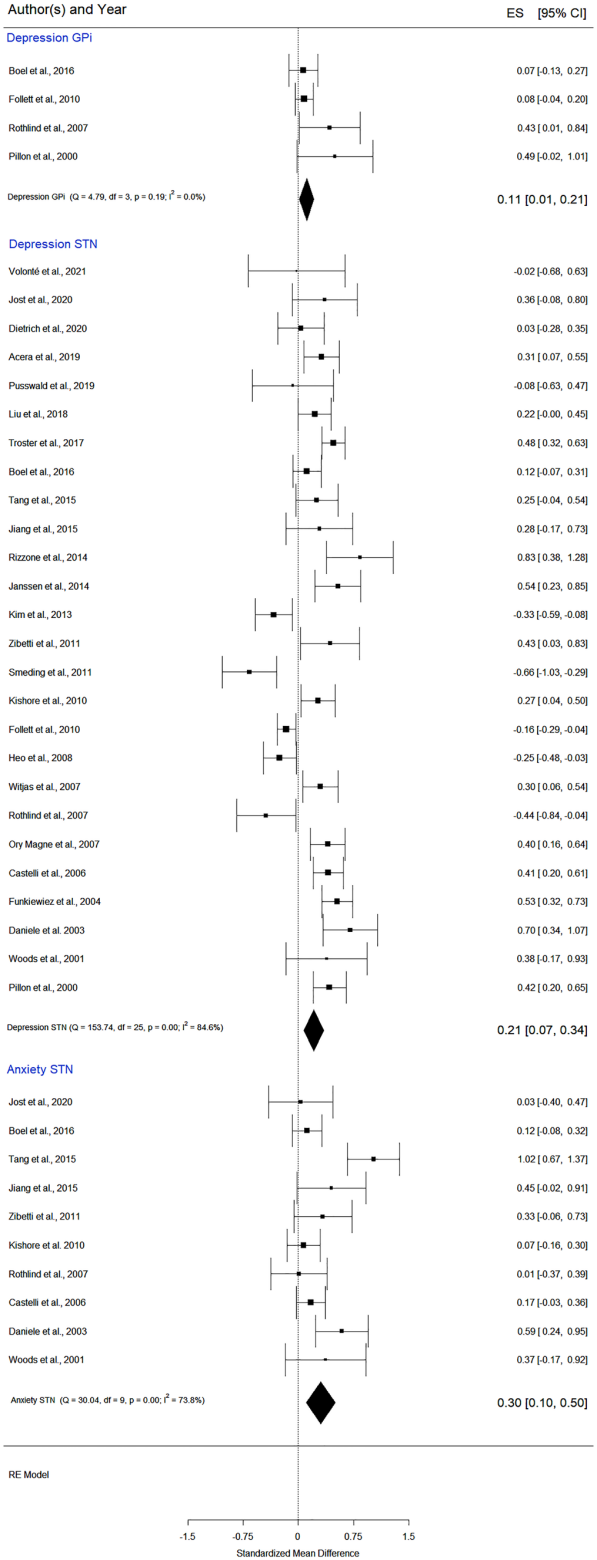
Forest plot – DBS effects on mood: depression and anxiety

Analysis of DBS publications reporting data immediately before and after treatment (12 to 36 months follow-up) revealed a statistically significant but very small SMD of 0,34 (95% CI = [0,04, 0,65]; p = 0,02; K = 27, N = 1512) *for STN* (in Appendix A Figs. S29, S30 and S31, and in the manuscript Fig. S32) and a SMD of 0,11 (95% CI = [0,01, 0,21]; p = 0,03; K = 4, N = 231) for *GPi stimulation* (Fig. 7, in Appendix A Figs. S32 and S33), suggesting an improvement after DBS. Schuepbach et al. (2013) study was identified as a potential outlier. After its exclusion, the *effect size for STN* was of 0,21 (95% CI = [0,07; 0,34]; p = 0,002; K = 26, N = 1261), (Fig. 7).

Since the regression test for the GPi funnel plot asymmetry was statistically significant (z = 2,04, p = 0,04), we used the fill and trim method. The new data, with one imputed missing study (Table 3), indicate an SMD of 0,10 (p = 0,05; K = 5), a small effect size that barely reaches statistical significance. Overall, these results suggest that depression was slightly reduced at follow-up compared to pre-surgery.

#### Anxiety

The meta-analysis of 10 *STN DBS* studies showed a significant improvement after DBS (Fig. 7, and in Appendix A: Figs. S34 and S35), SMD of 0,30 (95% CI = [0,10; 0,50]; p = 0,01; K = 10, N = 290). Anxiety was assessed by means of the *State-Trait Anxiety Inventory—STAI* (Castelli et al., 2006; Rothlind et al., 2007; Zibetti et al., 2011), *the Beck Anxiety Inventory* (Tang et al., 2015; Woods et al., 2001), the Zung’s Anxiety Scale (Daniele et al., 2003), the *Hamilton Anxiety Scale* (Jiang et al., 2015), and the *Hospital Anxiety and Depression Scale* (Jost et al., 2021; Boel et al., 2016; Kishore et al., 2010). The Baujat plot (in Appendix A Fig. S34) and the Viechtbauer and Cheung influential test identified one study (Tang et al., 2015) as a potential outlier. From the forest plot (Fig. 7), it is evident that the effect size and confidence interval were higher than reported in the other publications. In conclusion, the current data indicate that after STN BDS, patients have slightly lower anxiety and depression levels.

### GRADE Assessment

The GRADE quality of evidence for all pre-post design outcomes (Tables 4) was low (i.e., the true effect may differ significantly from the estimate) due to several methodological issues: (i) cognitive decline can occur in PD patients over time independently of treatment, (ii) in serial neuropsychological assessments, an improved performance may result from practice effects, although the relatively long intervals between cognitive assessments should partially reduce this confounding factor, (iii) heterogeneity of patients’ groups, (iv) small sample size in some studies (Woods et al., 2001).

**Table 4:** GRADEpro summary: DBS in Parkinson's disease

**Certainty assessment**						**N° of patients**	**Effect**	**Certainty**
**№ of studies**	**Study design**	**Risk of bias**	**Inconsistency**	**Indirectness**	**Imprecision**		**(95% CI)**	
**Delayed Recall STN & GPi DBS** (follow up: range 1 years to 3 years)								
24	observational studies between (DBS vs. ODT) within (pre vs. post)	not serious	not serious	not serious	not serious	1429	SMD **0.13 SD lower** (0.23 lower to 0.02 lower)	⨁⨁◯◯ LOW
**Backward Digit Span STN & GPi DBS** (follow up: range 1 years to 3 years)								
9	observational studies between (DBS vs. ODT) within (pre vs. post)	not serious	not serious	not serious	not serious	156	SMD **0.11 SD higher** (0.02 lower to 0.23 higher)	⨁⨁◯◯ LOW
**Immediate Recall STN & GPi DBS** (follow up: range 1 years to 3 years)								
16	observational studies between (DBS vs. ODT) within (pre vs. post)	not serious	not serious	not serious	not serious	720	SMD **0.06 SD lower** (0.21 lower to 0.09 higher)	⨁⨁◯◯ LOW
**Phonemic fluency STN DBS** (follow up: range 1 years to 3 years)								
30	observational studies between (DBS vs. ODT) within (pre vs. post)	not serious	not serious	not serious	not serious	1308	SMD **0.4 SD lower** (0.49 lower to 0.32 lower)	⨁⨁◯◯ LOW
**Phonemic fluency GPi DBS** (follow up: range 1 years to 3 years)								
6	observational studies between (DBS vs. ODT) within (pre vs. post)	not serious	not serious	not serious	not serious	304	SMD **0.3 SD lower** (0.55 lower to 0.04 higher)	⨁⨁◯◯ LOW
**Stroop Color and Word STN DBS** (follow up: range 1 years to 3 years)								
21	observational studies between (DBS vs. ODT) within (pre vs. post)	not serious	not serious	not serious	not serious	958	SMD **0.3 SD lower** (0.39 lower to 0.22 lower)	⨁⨁◯◯ LOW
**Stroop Color and Word GPi DBS** (follow up: range 1 years to 3 years)								
6	observational studies between (DBS vs. ODT) within (pre vs. post)	not serious	not serious	not serious	not serious	304	SMD **0.16 SD lower** (0.38 lower to 0.05 higher)	⨁⨁◯◯ LOW
**Semantic Fluency STN DBS** (follow up: range 1 years to 3 years)								
28	observational studies between (DBS vs. ODT) within (pre vs. post)	not serious	not serious	not serious	not serious	1378	SMD **0.49 SD lower** (0.57 lower to 0.41 lower)	⨁⨁◯◯ LOW
**Semantic Fluency GPi DBS** (follow up: range 1 years to 3 years)								
6	observational studies between (DBS vs. ODT) within (pre vs. post)	not serious	not serious	not serious	not serious	304	SMD **0.5 SD lower** (0.59 lower to 0.4 lower)	⨁⨁◯◯ LOW
**Depression STN DBS** (follow up: range 1 years to 3 years)								
26	observational studies between (DBS vs. ODT) within (pre vs. post)	not serious	not serious	not serious	not serious	1261	SMD **0.2 SD higher** (0.04 higher to 0.36 higher)	⨁⨁◯◯ LOW
**Depression GPi DBS** (follow up: range 1 years to 3 years)								
4	observational studies between (DBS vs. ODT) within (pre vs. post)	not serious	not serious	not serious	serious ^a^	231	SMD **0.11 SD higher** (0.01 higher to 0.21 higher)	⨁◯◯◯ VERY LOW
**Anxiety STN DBS** (follow up: range 1 years to 3 years)								
10	observational studies between (DBS vs. ODT) within (pre vs. post)	not serious	not serious	not serious	not serious	290	SMD **0.3 SD higher** (0.1 higher to 0.5 higher)	⨁⨁◯◯ LOW

*CI* Confidence interval, *SMD* Standardized mean difference (Hedges’g), *STN* subthalamic nucleus, *GPi* globus pallidus pars interna, *DBS* deep brain stimulation, *ODT* optimal drug therapy (control group)

**GRADE Working Group grades of evidence**

High quality: Further research is very unlikely to change our confidence in the estimate of effect

Moderate quality: The authors believe that the true effect is probably close to the estimated effect

Low quality: Further research is very likely to have an important impact on our confidence in the estimated effect and could change the estimate

Very low quality: We are very uncertain about the estimate

**Explanations**

^a^A very small number of included studies

The level of evidence for study designs that included a control group (Table 5) was moderate (i.e., the true effect is likely to be close to the estimated effect, but it is still possible to be different). The downgrade was due to the lack of randomization, possible publication bias, and because the no-DBS and DBS PD groups were not perfectly matched, especially regarding disease duration that was shorter in the no-DBS PD group (Table 2).

**Table 5:** GRADEpro summary: DBS compared to ODT in Parkinson's disease

**Certainty assessment**												**№ of patients**				**Effect**		**Certainty**
**N° of studies**		**Study design**		**Risk of bias**		**Inconsistency**		**Indirectness**		**Imprecision**		**DBS**		**NO DBS**		**(95% CI)**		
**Delayed Recall (DBS vs. ODT)**—follow up: range 1 years to 3 years																		
5		randomized trials		serious ^a^		not serious		not serious		not serious		200		130		SMD **0.4 SD lower** (0.75 lower to 0.05 lower)		⨁⨁⨁◯ MODERATE
**Phonemic fluency (DBS vs. PD)**—follow up: range 1 years to 3 years																		
8		randomized trials		serious ^a^		not serious		not serious		not serious		256		183		SMD **0.56 SD lower** (0.79 lower to 0.33 lower)		⨁⨁⨁◯ MODERATE
**Stroop Color and Word (DBS vs. PD)**—follow up: range 1 years to 3 years																		
5		randomized trials		serious ^a^		not serious		not serious		not serious		182		117		SMD **0.45 SD lower** (0.74 lower to 0.15 lower)		⨁⨁⨁◯ MODERATE
**Semantic Fluency (DBS vs. PD)**—follow up: range 1 years to 3 years																		
7	randomized trials		serious ^a^		not serious		not serious		not serious		217		139		SMD **0.53 SD lower** (0.78 lower to 0.29 lower)		⨁⨁⨁◯ MODERATE	

*ODT*, optimal drug therapy (control group), *CI* Confidence interval, *SMD* Standardized mean difference (Hedges’g), *STN* subthalamic nucleus, *GPi* globus pallidus pars interna, *DBS* deep brain stimulation

**GRADE Working Group grades of evidence**

High quality: Further research is very unlikely to change our confidence in the estimate of effect

Moderate quality: The authors believe that the true effect is probably close to the estimated effect

Low quality: Further research is very likely to have an important impact on our confidence in the estimated effect and could change the estimate

Very low quality: We are very uncertain about the estimate

**Downgraded explanations**

^a^Downgraded as there were serious limitations identified in the risk of bias, since not all the studies were RCTs

## Discussion

STN and GPi DBS are effective, accepted therapies for PD motor complications, especially when drugs are not effective (Rughani et al., 2018). While DBS has been found to improve motor symptoms (Mao et al., 2019), long-term decrease in cognitive function has been reported (Merola et al., 2014). In terms of neuropsychological performance, the effects of DBS in PD have been investigated in several studies and meta-analyses (Altinel et al., 2019; Castrioto et al., 2014; Combs et al., 2015, 2018; Elgebaly et al., 2018; Liu et al., 2014; Mansouri et al., 2018; Martínez-Martínez et al., 2017; Parsons et al., 2006; Vizcarra et al., 2019). The number of reviews and meta-analyses on this topic is large when compared to the number of actual high quality, randomized clinical trials (or experimental papers), pointing to the difficulty of creating an experimental setting and controlling for confounding variables.

Our meta-analysis differs from the previous ones in three aspects. (i) The analyses concerned only four domains (memory, executive functions, language, and emotional state) that were previously reported as crucial for the PD DBS (Dujardin et al., 2016; Papagno & Trojano, 2018; Trojano & Papagno, 2018), and we tried to select homogeneous neuropsychological tools (the most frequently used neuropsychological tests). We chose this procedure as different tests might target different aspects of the same cognitive function. Previous publications consider the same function explored with different tools (Combs et al., 2015; Elgebaly et al., 2018; Wu et al., 2014). (ii) Besides the strict selection of neuropsychological tests, an additional novelty is a clear follow-up time-point (between 12- and 36-months post-DBS). Because the number of published RCTs with PD patients is small and the methodological quality is lowered by the difficulty to balance the DBS with the control group, we chose a specific follow-up period. In fact, in most cases, DBS patients have a longer period of disease and are older than no-DBS PD participants. We considered the 12 to 36 months post DBS period to be optimal for two main reasons: a) it is long enough to exclude immediate post-surgery effects allowing a post-intervention recovery, and b) it is short enough to avoid a cognitive decline due to disease progression or ageing. Previous meta-analyses did not control for the follow-up duration (e.g., 3-months, 6-months, 1-year, 6-years, etc. were considered together) (Sako et al., 2014; Wang et al., 2016; Xie et al., 2016). This creates an important bias since the cognitive performance at three months from the DBS might be different from the one observed at 12 months. Moreover, ON verses OFF stimulation papers were not differentiated either, while we only considered DBS ON. The restriction on the follow-up period has reduced the number of included studies but has offered a better perspective of the DBS's long-term effects on cognition. When possible, we compared DBS to PD control group. Unfortunately, the number of included papers was exceedingly small (four to eight) and, as mentioned, not matched to each other. (iii) Our data are an update of the previous reviews, including very recent publications (Volonté et al., 2021) and more than 2000 PD DBS participants.

Results showed that delayed recall (long-term verbal and visuospatial) performance after DBS significantly changed in the 1–3-year follow-up. The decline was more relevant in the DBS group as compared to the ODT group (Hedges’ g = -0, 40; p = 0,02) but also in the pre- verses post-DBS testing (Hedges’ g = -0,13, p = 0,02). This decline is reported in several studies (Mehanna et al., 2017; Nassery et al., 2016; Xie et al., 2016). Because it co-occurs with the decline of other functions, the delayed memory changes could depend on executive function and processing speed deterioration (Higginson et al., 2003).

The immediate recall analysis showed no statistically significant changes after one to three years post DBS (Hedges' g = -0,06; p = 0,645). This is in contrast with previous reviews that found a negative change in memory performance. To our knowledge, the meta-analyses that indicated a subtle decline in memory after DBS aggregated both delay and immediate recall data, so the observed changes might have been influenced by the delayed recall performance (Xie et al., 2016; Wang et al, 2016, Elgebaly et al., 2018; Parsons et al., 2006).

Working memory impairment is often encountered in PD patients (Papagno & Trojano, 2018), and one would predict that surgery, such as DBS, could further disrupt this function. Our results, similar with the ones published by Martínez-Martínez et al., (2017) indicated a small, statistically insignificant, effect size (Hedges' g = 0,11; p = 0,09). We can conclude that there is no long-term negative impact of DBS on the working memory, at least as measured by the backward digit span.

Executive Functions and Attention impairments are among the most consistent findings in PD natural progression (Papagno & Trojano, 2018) and are also described after DBS (Martínez-Martínez et al., 2017). A large number of cognitive tests are subsumed under the executive functions heading, but we selected only two tests consistently applied in the literature that better capture flexibility and inhibition. It was not our intention to cover all aspects of executive functions. In a future meta-analysis, the focus can be exclusively on executive functions, and one can cover all aspects from attention to working memory and problem solving. A decrease in performance was observed in both the phonemic verbal fluency and Stroop Test (DBS versus control, Hedges’ g = -0,45; p = 0,003), while no significant changes were observed after GPi stimulation on the Stroop Test (Hedges’ g =—0,16; p = 0,13). It has been suggested that the effects on phonological verbal fluency could depend on the position of the electrodes over the left STN (York et al., 2009), PD progression (Muslimović et al., 2007), or surgery microlesions’ effects (Lefaucheur et al., 2012; Mehanna et al., 2017; Wang et al., 2016). It has also been found that DBS can reduce left temporal and inferior frontal cortex activity, thus interfering with verbal fluency (Fasano et al., 2012). Another valid hypothesis could be that disease progression is associated with cognitive deterioration, especially in patients who would be DBS candidates. Meta-analyses that compared STN and GPi stimulation effects on cognition indicate that the electrodes’ locations might be crucial in order to minimize side effects. More specifically, some authors suggest that GPi might be safer than STN and that unilateral stimulation might be preferable to bilateral stimulation (Elgebaly et al., 2018; Liu et al., 2014). However, additional factors, such as the area of active stimulation or the volume of the electrode contact, can affect the outcome. In our review, we could not control the stimulation type, bilateral vs. unilateral, due to the small number of studies applying unilateral DBS, but, when possible, we differentiated the stimulation target. We found a difference in the Stroop test with the STN group, but not the GPi group, showing decreased performance. In conclusion, there was some decrease in verbal fluency and inhibition after DBS.

Regarding linguistic abilities, although changes in verbal fluency are common in PD patients (Muslimović et al., 2007), the decrease after DBS is more severe. While Elgebaly et al. (2018) identified a slight improvement in the GPi DBS group in verbal fluency, our data show a moderate decrease in both semantic and phonemic fluency performance even with GPi stimulation (e.g., semantic verbal fluency GPi DBS: Hedges’ g = -0,50; p = < 0,0001).

An improvement (small to medium effect sizes) of depression (SMD of 0,34; p = 0,02 for STN and a SMD of 0,11; p = 0,03 for GPi stimulation), and anxiety (SMD of 0,30; p = 0,01) was found. It is hard to interpret pre versus post data in terms of causality without a control group. For instance, it is plausible that emotions related to the upcoming intervention influenced pre-DBS anxiety and depression scores or that the improvement of motor symptoms after DBS reduced depression. However, there is also evidence indicating no significant difference between the DBS group and the medically treated group (Nassery et al., 2016). Besides anxiety and depression, another mood change increasingly recognized post-DBS, although not investigated in this review, is apathy (commonly described as loss of motivation, decreased initiative and energy, and an emotional indifference). Two recent meta-analyses (Wang et al., 2018; Zoon et al., 2021) concluded that apathy was more prevalent after STN DBS compared to the pre-operative state or to control groups managed only with medication. No data are available for social cognition, which is another area of impairment in PD (Mattavelli et al., 2021).

We must acknowledge several limitations in the present meta-analysis. As with all meta-analyses, the quality is limited by the number and the level of the included studies (GRADE analysis). Standardized tests are not always used, and some of them are performed in different versions (e.g., memory tests). Often it is not clearly reported how the final scores were calculated, making it hard to properly choose and group the correct means and SD for our purpose. There is also the risk of publication bias, meaning that studies with significant findings are more likely to be published with an overestimation of the effects. In order to control for this last limitation, as reported, we conducted publication bias analysis and when necessary, we corrected for the missing studies applying the trim and fill method. Reviews are also prone to search and selection bias. Based on the current findings and given the low statistical power of the pooled analysis, especially regarding the control group studies, it is clear that further RCTs comparing DBS and PD control groups (best pharmacological treatment) are required, with standardized outcome measures and adequately reported results. An important obstacle in planning RCTs is the patients' reluctance to participate in randomized studies. They are often unwilling to be in a control group for more than a few months. This is especially relevant now that many DBS devices have been approved and surgical centers have proliferated (i.e., patients can access DBS procedures without the onerous demands involved in research trials). The other issue is that control groups in long-term studies have not been constituted by random assignment and provide a false sense of security that extraneous variables are being controlled for.

We acknowledge that variables such as L-dopa response or age and attention at baseline are predictors of cognitive and psychosocial outcome after DBS (Smeding et al., 2011). Unfortunately, this type of data is not always reported, but in Table 2 we have included a selection of variables regarding the participants’ characteristics which may help the reader gain an understanding of the factors that may influence DBS cognitive outcomes.

Summing up, our findings add new data to the existing literature by demonstrating that cognition and emotion show significant changes after DBS, some positive, such as a decrease in anxiety and depression, and some negative, such as impairment in long-term memory, verbal fluency and specific subdomains of executive functions, for example, flexibility and inhibition.

These results have a possible simple explanation, since both GPi and STN are part of a circuit involved in inhibition or disinhibition of frontal areas (Huh et al., 2018). What can be taken for granted is that verbal fluency, long-term memory, and inhibitory control should be intact before submitting a patient to DBS, being crucial aspects to test before treatment. In line with this perspective, a recent publication presented data suggesting that poor presurgical performance in verbal memory recognition, language processing, and visuospatial performance is associated with patient- or caregiver-reported decline following DBS surgery (Mills et al., 2019). Of course, this is not a strict and forward recommendation especially because the GRADE evaluation indicates that new publications could modify the observed effects. Clinicians should carefully balance the potential benefits and risks of a DBS intervention based upon each patient’s characteristics.

Finally, the improvement of motor symptoms probably produces a better perception of quality of life, even in presence of cognitive worsening (Mehanna et al., 2017; Merola et al., 2014; Wu et al., 2014).

## Supplementary Information

Below is the link to the electronic supplementary material.

Supplementary file1 (DOCX 585 KB)

## Data Availability

In Appendix A. Supplementary data.
