# Supplementary material for: Deep Brain Stimulation in Parkinson Disease: A Meta-analysis of the Long-term Neuropsychological Outcomes
Source: Neuropsychol Rev. 2022 Mar 23;33(2):307–46. doi: 10.1007/s11065-022-09540-9 (PMC10148791; doi:10.1007/s11065-022-09540-9)

**Appendix A (supplementary data)**

**Deep Brain Stimulation in Parkinson Disease: a meta-analysis of the long-term neuropsychological outcomes**

**Neuropsychology Review**

Madalina Bucur^a^ and Costanza Papagno ^a,b*^

^a^ Center for Mind/Brain Sciences (CIMeC), University of Trento, Italy

^b^ Department of Psychology, University of Milano-Bicocca

*** Corresponding author:**

Costanza Papagno, MD, PhD

CeRiN (Center for Cognitive Neurorehabilitation)

CIMeC, University of Trento

Via Matteo del Ben 5/b

38068 Rovereto (TN)

Italy

e-mail: costanza.papagno@unitn.it

tel 0464 808165

ORCID ID: 0000-0002-3659-6294

**Appendix A - Materials and methods**

**Tables legends:**

Table S1. | Summary of the Physiotherapy Evidence Database (PEDro)

Table S2. | Risk of bias

**Figures and captions:**

Fig. S1 Baujat plot - DBS effects on delayed recall (overall)

Fig. S2 Funnel plot - DBS effects on delayed recall (overall)

Fig. S3 Baujat plot - DBS vs. ODT PD effects on delayed recall

Fig. S4 Funnel plot - DBS vs. ODT PD effects on delayed recall

Fig. S5 Baujat plot - DBS effects on backward digit span (overall)

Fig. S6 Funnel plot - DBS effects on backward digit span (overall)

Fig. S7 Baujat plot - DBS effects on immediate recall (overall)

Fig. S8 Funnel plot - DBS effects on immediate recall (overall)

Fig. S9 Baujat plot – GPi DBS effects on phonemic verbal fluency (overall)

Fig. S10 Funnel plot – GPi DBS effects on phonemic verbal fluency (overall)

Fig. S11 Baujat plot – DBS vs. ODT PD effects on phonemic verbal fluency

Fig. S12 Funnel plot – DBS vs. ODT PD effects on phonemic verbal fluency

Fig. S13 Baujat plot – STN DBS effects on Stroop test (color–word) (overall)

Fig. S14 Funnel plot – STN DBS effects on Stroop test (color–word) (overall)

Fig. S15 Baujat plot – GPi DBS effects on Stroop test (color–word) (overall)

Fig. S16 Funnel plot – GPi DBS effects on Stroop test (color–word) (overall)

Fig. S17 Baujat plot – DBS vs. ODT PD effects on Stroop test (color–word)

Fig. S18 Funnel plot – DBS vs. ODT PD effects on Stroop test (color–word)

Fig. S19 Baujat plot – STN DBS effects on semantic verbal fluency (overall)

Fig. S20 Funnel plot – STN DBS effects on semantic verbal fluency (overall)

Fig. S21 Baujat plot – GPi DBS effects on semantic verbal fluency (overall)

Fig. S22 Funnel plot – GPi DBS effects on semantic verbal fluency (overall)

Fig. S23 Baujat plot – DBS vs. ODT PD effects on semantic verbal fluency

Fig. S24 Funnel plot – DBS vs. ODT PD effects on semantic verbal fluency

Fig. S25 Forest plot – STN DBS effects on depression (overall) before Schuepbach et al. (2013)’s exclusion

Fig. S26 Baujat plot – STN DBS effects on depression (overall)

Fig. S27 Funnel plot – STN DBS effects on depression (overall)

Fig. S28 Baujat plot – GPi DBS effects on depression (overall)

Fig. S29 Funnel plot – GPi DBS effects on depression (overall)

Fig. S30 Baujat plot – STN DBS effects on anxiety (overall)

Fig. S31 Funnel plot – STN DBS effects anxiety (overall)

**4. Meta-analysis results:**

Table S1 Summary of the Physiotherapy Evidence Database (PEDro)

| **Nr.** | **Study** | **1. eligibility criteria** | **2. randomized allocation** | **3. concealed allocation** | **4. comparable at baseline** | **5. blinded subjects** | **6. blinded therapists** | **7. blinded assessors** | **8. adequate follow-up** | **9. intention to treat analysis** | **10. between-group comparison** | **11. point estimates and variability** | **Total** |
| --- | --- | --- | --- | --- | --- | --- | --- | --- | --- | --- | --- | --- | --- |
| 1. | Pillon et al. 2000 | 1 | 0 | 0 | 0 | 0 | 0 | 0 | 1 | 1 | 0 | 1 | 3 |
| 2. | Dujardin et al. 2001 | 1 | 0 | 0 | 0 | 0 | 0 | 0 | 1 | 1 | 0 | 1 | 3 |
| 3. | Woods et al. 2001 | 1 | 0 | 0 | 0 | 0 | 0 | 0 | 1 | 1 | 0 | 1 | 3 |
| 4. | Daniele et al. 2003 | 1 | 0 | 0 | 0 | 0 | 0 | 0 | 1 | 1 | 0 | 1 | 3 |
| 5. | Moretti et al. 2003 | 1 | 0 | 0 | 1 | 0 | 0 | 0 | 1 | 1 | 1 | 1 | 5 |
| 6. | Funkiewiez et al. 2004 | 1 | 0 | 0 | 0 | 0 | 0 | 0 | 1 | 1 | 0 | 1 | 3 |
| 7. | Smeding et al. 2005 | 1 | 0 | 0 | 0 | 0 | 0 | 0 | 1 | 1 | 0 | 1 | 3 |
| 8. | Castelli et al. 2006 | 1 | 0 | 0 | 0 | 0 | 0 | 0 | 1 | 1 | 0 | 1 | 3 |
| 9. | Cilia et al. 2007 | 1 | 0 | 0 | 1 | 0 | 0 | 0 | 1 | 1 | 1 | 1 | 5 |
| 10. | Contarino et al. 2007 | 1 | 0 | 0 | 0 | 0 | 0 | 0 | 1 | 1 | 0 | 1 | 3 |
| 11. | Klempirova et al. 2007 | 1 | 0 | 0 | 0 | 0 | 0 | 0 | 1 | 1 | 0 | 1 | 3 |
| 12. | Ory‐Magne et al. 2007 | 1 | 0 | 0 | 0 | 0 | 0 | 0 | 1 | 1 | 0 | 1 | 3 |
| 13. | Rothlind et al. 2007 | 1 | 0 | 0 | 0 | 0 | 0 | 0 | 1 | 1 | 0 | 1 | 3 |
| 14. | Witjas et al. 2007 | 1 | 0 | 0 | 0 | 0 | 0 | 0 | 1 | 1 | 0 | 1 | 3 |
| 15. | Fraraccio et al. 2008 | 1 | 0 | 0 | 0 | 0 | 0 | 0 | 1 | 1 | 0 | 1 | 3 |
| 16. | Heo et al. 2008 | 1 | 0 | 0 | 0 | 0 | 0 | 0 | 1 | 1 | 0 | 1 | 3 |
| 17. | Zangaglia et al. 2009 | 1 | 0 | 0 | 1 | 0 | 0 | 0 | 1 | 1 | 1 | 1 | 5 |
| 18. | Castelli et al. 2010 | 1 | 0 | 0 | 1 | 0 | 0 | 0 | 1 | 1 | 1 | 1 | 5 |
| 19. | Follett et al. 2010 | 1 | 0 | 0 | 0 | 0 | 0 | 0 | 1 | 1 | 0 | 1 | 3 |
| 20. | Kishore et al., 2010 | 1 | 0 | 0 | 1 | 0 | 1 | 0 | 1 | 1 | 0 | 1 | 5 |
| 21. | Mikos et al. 2010 | 1 | 0 | 0 | 1 | 0 | 0 | 0 | 1 | 1 | 1 | 1 | 5 |
| 22. | Smeding et al. 2011 | 1 | 0 | 0 | 1 | 0 | 0 | 0 | 1 | 1 | 1 | 1 | 5 |
| 23. | Williams et al. 2011 | 1 | 0 | 0 | 1 | 0 | 0 | 0 | 1 | 1 | 1 | 1 | 5 |
| 24. | Zibetti et al. 2011 | 1 | 0 | 0 | 0 | 0 | 0 | 0 | 1 | 1 | 0 | 1 | 3 |
| 25. | Sjöberg et al. 2012 | 1 | 0 | 0 | 0 | 0 | 0 | 0 | 1 | 1 | 0 | 1 | 3 |
| 26. | Yamanaka et al. 2012 | 1 | 0 | 0 | 0 | 0 | 0 | 0 | 1 | 1 | 0 | 1 | 3 |
| 27. | Kim et al. 2013 | 1 | 0 | 0 | 0 | 0 | 0 | 0 | 1 | 1 | 0 | 1 | 3 |
| 28. | Schuepbach et al., 2013 | 1 | 1 | 0 | 1 | 0 | 0 | 1 | 1 | 1 | 1 | 1 | 7 |
| 29. | Asahi et al. 2014 | 1 | 0 | 0 | 0 | 0 | 0 | 0 | 1 | 1 | 0 | 1 | 3 |
| 30. | Janssen et al. 2014 | 1 | 0 | 0 | 0 | 0 | 0 | 0 | 1 | 1 | 0 | 1 | 3 |
| 31. | Merola et al. 2014 | 1 | 0 | 0 | 0 | 0 | 0 | 0 | 1 | 1 | 0 | 1 | 3 |
| 32. | Rizzone et al. 2014 | 1 | 0 | 0 | 0 | 0 | 0 | 0 | 1 | 1 | 0 | 1 | 3 |
| 33. | Jiang et al. 2015 | 1 | 0 | 0 | 0 | 0 | 0 | 0 | 1 | 1 | 0 | 1 | 3 |
| 34. | Odekerken et al. 2015 | 1 | 0 | 0 | 0 | 0 | 0 | 0 | 1 | 1 | 0 | 1 | 3 |
| 35. | Tang et al. 2015 | 1 | 0 | 0 | 0 | 0 | 0 | 0 | 1 | 1 | 0 | 1 | 3 |
| 36. | Tramontana et al. 2015 | 1 | 1 | 0 | 1 | 0 | 1 | 1 | 1 | 1 | 1 | 1 | 8 |
| 37. | Boel et al. 2016 | 1 | 0 | 0 | 0 | 0 | 0 | 0 | 1 | 1 | 0 | 1 | 3 |
| 38. | Tröster et al., 2017 | 1 | 0 | 0 | 0 | 0 | 0 | 0 | 1 | 1 | 0 | 1 | 3 |
| 39. | Foki et al. 2018 | 1 | 0 | 0 | 1 | 0 | 0 | 0 | 1 | 1 | 1 | 1 | 5 |
| 40. | Acera et al. 2019 | 1 | 0 | 0 | 0 | 0 | 0 | 0 | 1 | 1 | 0 | 1 | 3 |
| 41. | Liu et al., 2019 | 1 | 0 | 0 | 0 | 0 | 0 | 0 | 1 | 1 | 0 | 1 | 3 |
| 42. | Pusswald et al., 2019 | 1 | 0 | 0 | 1 | 0 | 0 | 0 | 1 | 1 | 1 | 1 | 5 |
| 43. | Dietrich et al., 2020 | 1 | 0 | 0 | 0 | 0 | 0 | 0 | 1 | 1 | 0 | 1 | 3 |
| 44. | Jost et al., 2020 | 1 | 0 | 0 | 1 | 0 | 0 | 0 | 1 | 1 | 1 | 1 | 5 |
| 45. | Leimbach et al., 2020 | 1 | 0 | 0 | 1 | 0 | 0 | 0 | 1 | 1 | 1 | 1 | 5 |
| 46. | Mulders et al., 2020 | 1 | 0 | 0 | 0 | 0 | 0 | 0 | 1 | 1 | 0 | 1 | 3 |
| 47. | You et al., 2020 | 1 | 0 | 0 | 1 | 0 | 0 | 0 | 1 | 1 | 1 | 1 | 5 |
| 48. | Volonté et al., 2021 | 1 | 0 | 0 | 0 | 0 | 0 | 0 | 1 | 1 | 0 | 1 | 3 |
|  |  |  |  |  |  |  |  |  |  |  |  |  |  |
|  | **Yes = 1** |  |  |  |  |  |  |  |  |  |  |  |  |
|  | **No = 0** |  |  |  |  |  |  |  |  |  |  |  |  |

Table S2 Risk of bias

| **Nr.** |  | **Design** | **random sequence generation** | **allocation concealment** | **blinding of participants and personnel** | **blinding of outcome assessment** | **objective outcome** | **more than (80%) of participants enrolled in trials were included in the analysis** | **no reporting bias** | **potential of other bias** | **the trials end as scheduled** | **total** |
| --- | --- | --- | --- | --- | --- | --- | --- | --- | --- | --- | --- | --- |
| 1. | Pillon et al. 2000 | within  (pre vs. post) | 0 | 0 | 0 | 0 | 1 | 1 | 1 | 0 | 1 | 4 |
| 2. | Dujardin et al. 2001 | within  (pre vs. post) | 0 | 0 | 0 | 0 | 1 | 1 | 1 | 0 | 1 | 4 |
| 3. | Woods et al. 2001 | within  (pre vs. post) | 0 | 0 | 0 | 0 | 1 | 1 | 1 | 0 | 1 | 4 |
| 4. | Daniele et al. 2003 | ABBA design within (pre vs. post) | 0 | 0 | 0 | 0 | 1 | 1 | 1 | 0 | 1 | 4 |
| 5. | Moretti et al. 2003 | between (DBS vs control) and within (pre vs. post) | 0 | 0 | 0 | 0 | 1 | 1 | 1 | 0 | 1 | 4 |
| 6. | Funkiewiez et al. 2004 | within  (pre vs. post) | 0 | 0 | 0 | 0 | 1 | 1 | 1 | 0 | 1 | 4 |
| 7. | Smeding et al. 2005 | within  (pre vs. post) | 0 | 0 | 0 | 0 | 1 | 1 | 1 | 0 | 1 | 4 |
| 8. | Castelli et al. 2006 | within  (pre vs. post) | 0 | 0 | 0 | 0 | 1 | 1 | 1 | 0 | 1 | 4 |
| 9. | Cilia et al. 2007 | between (DBS vs control) and within (pre vs. post) | 0 | 0 | 0 | 0 | 1 | 1 | 1 | 0 | 1 | 4 |
| 10. | Contarino et al. 2007 | within  (pre vs. post) | 0 | 0 | 0 | 0 | 1 | 1 | 1 | 0 | 1 | 4 |
| 11. | Klempirova et al. 2007 | within  (pre vs. post) | 0 | 0 | 0 | 0 | 1 | 1 | 1 | 0 | 1 | 4 |
| 12. | Ory‐Magne et al. 2007 | within  (pre vs. post) | 0 | 0 | 0 | 0 | 1 | 1 | 1 | 0 | 1 | 4 |
| 13. | Rothlind et al. 2007 | within  (pre vs. post) | 0 | 0 | 0 | 0 | 1 | 1 | 1 | 0 | 1 | 4 |
| 14. | Witjas et al. 2007 | within  (pre vs. post) | 0 | 0 | 0 | 0 | 1 | 1 | 1 | 0 | 1 | 4 |
| 15. | Fraraccio et al. 2008 | within  (pre vs. post) | 0 | 0 | 0 | 0 | 1 | 1 | 1 | 0 | 1 | 4 |
| 16. | Heo et al. 2008 | within  (pre vs. post) | 0 | 0 | 0 | 0 | 1 | 1 | 1 | 0 | 1 | 4 |
| 17. | Zangaglia et al. 2009 | between (DBS vs control) and within (pre vs. post) | 0 | 0 | 0 | 0 | 1 | 1 | 1 | 0 | 1 | 4 |
| 18. | Castelli et al. 2010 | between (DBS vs control) and within (pre vs. post) | 0 | 0 | 0 | 0 | 1 | 1 | 1 | 0 | 1 | 4 |
| 19. | Follett et al. 2010 | within  (pre vs. post) | 0 | 0 | 0 | 0 | 1 | 1 | 1 | 0 | 1 | 4 |
| 20. | Kishore et al., 2010 | within  (pre vs. post) | 0 | 0 | 0 | 1 | 1 | 1 | 1 | 0 | 1 | 5 |
| 21. | Mikos et al. 2010 | between (DBS vs control) and within (pre vs. post) | 0 | 0 | 0 | 0 | 1 | 1 | 1 | 0 | 1 | 4 |
| 22. | Smeding et al. 2011 | between (DBS vs control) and within (pre vs. post) | 0 | 0 | 0 | 0 | 1 | 1 | 1 | 0 | 1 | 4 |
| 23. | Williams et al. 2011 | between (DBS vs control) and within (pre vs. post)) | 0 | 0 | 0 | 0 | 1 | 1 | 1 | 0 | 1 | 4 |
| 24. | Zibetti et al. 2011 | within  (pre vs. post) | 0 | 0 | 0 | 0 | 1 | 1 | 1 | 0 | 1 | 4 |
| 25. | Sjöberg et al. 2012 | within  (pre vs. post) | 0 | 0 | 0 | 0 | 1 | 1 | 1 | 0 | 1 | 4 |
| 26. | Yamanaka et al. 2012 | within  (pre vs. post) | 0 | 0 | 0 | 0 | 1 | 1 | 1 | 0 | 1 | 4 |
| 27. | Kim et al. 2013 | within  (pre vs. post) | 0 | 0 | 0 | 0 | 1 | 1 | 1 | 0 | 1 | 4 |
| 28. | Schuepbach et al., 2013 | between RCT (DBS vs control) and within (pre vs. post) | 1 | 0 | 0 | 1 | 1 | 1 | 1 | 0 | 1 | 6 |
| 29. | Asahi et al. 2014 | within  (pre vs. post) | 0 | 0 | 0 | 0 | 1 | 1 | 1 | 0 | 1 | 4 |
| 30. | Janssen et al. 2014 | within  (pre vs. post) | 0 | 0 | 0 | 0 | 1 | 1 | 1 | 0 | 1 | 4 |
| 31. | Merola et al. 2014 | within  (pre vs. post) | 0 | 0 | 0 | 0 | 1 | 1 | 1 | 0 | 1 | 4 |
| 32. | Rizzone et al. 2014 | within  (pre vs. post) | 0 | 0 | 0 | 0 | 1 | 1 | 1 | 0 | 1 | 4 |
| 33. | Jiang et al. 2015 | within  (pre vs. post) | 0 | 0 | 0 | 0 | 1 | 1 | 1 | 0 | 1 | 4 |
| 34. | Odekerken et al. 2015 | within  (pre vs. post) | 0 | 0 | 0 | 0 | 1 | 1 | 1 | 0 | 1 | 4 |
| 35. | Tang et al. 2015 | within  (pre vs. post) | 0 | 0 | 0 | 0 | 1 | 1 | 1 | 0 | 1 | 4 |
| 36. | Tramontana et al. 2015 | between RCT (DBS vs control) and within (pre vs. post) | 1 | 0 | 0 | 1 | 1 | 1 | 1 | 0 | 1 | 6 |
| 37. | Boel et al. 2016 | within  (pre vs. post) | 0 | 0 | 0 | 0 | 1 | 1 | 1 | 0 | 1 | 4 |
| 38. | Tröster et al., 2017 | within  (pre vs. post) | 0 | 0 | 0 | 0 | 1 | 1 | 1 | 0 | 1 | 4 |
| 39. | Foki et al. 2018 | between (DBS vs control) and within (pre vs. post) | 0 | 0 | 0 | 0 | 1 | 1 | 1 | 0 | 1 | 4 |
| 40. | Acera et al. 2019 | within  (pre vs. post) | 0 | 0 | 0 | 0 | 1 | 1 | 1 | 0 | 1 | 4 |
| 41. | Liu et al., 2019 | within  (pre vs. post) | 0 | 0 | 0 | 0 | 1 | 1 | 1 | 0 | 1 | 4 |
| 42. | Pusswald et al., 2019 | between (DBS vs control) and within (pre vs. post) | 0 | 0 | 0 | 0 | 1 | 1 | 1 | 0 | 1 | 4 |
| 43. | Dietrich et al., 2020 | within  (pre vs. post) | 0 | 0 | 0 | 0 | 1 | 1 | 1 | 0 | 1 | 4 |
| 44. | Jost et al., 2020 | between (DBS vs control) and within (pre vs. post) | 0 | 0 | 0 | 0 | 1 | 1 | 1 | 0 | 1 | 4 |
| 45. | Leimbach et al., 2020 | between (DBS vs control) and within (pre vs. post) | 0 | 0 | 0 | 0 | 1 | 1 | 1 | 0 | 1 | 4 |
| 46. | Mulders et al., 2020 | within  (pre vs. post) | 0 | 0 | 0 | 0 | 1 | 1 | 1 | 0 | 1 | 4 |
| 47. | You et al., 2020 | between (DBS vs control) and within (pre vs. post) | 0 | 0 | 0 | 0 | 1 | 1 | 1 | 0 | 1 | 4 |
| 48. | Volonté et al., 2021 | Within  (pre vs. post) | 0 | 0 | 0 | 0 | 1 | 1 | 1 | 0 | 1 | 4 |

**4.1. MEMORY**

- **Delayed Recall**

Fig. S1. Baujat plot - DBS effects on delayed recall (overall)


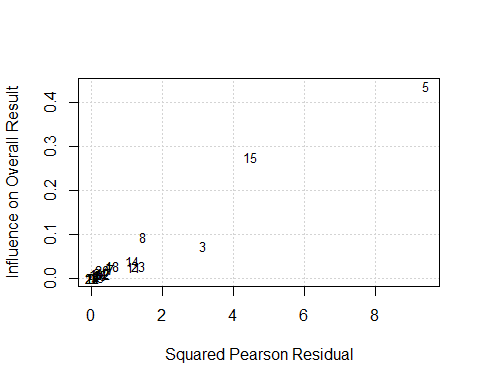


Fig. S2 Funnel plot - DBS effects on delayed recall (overall)


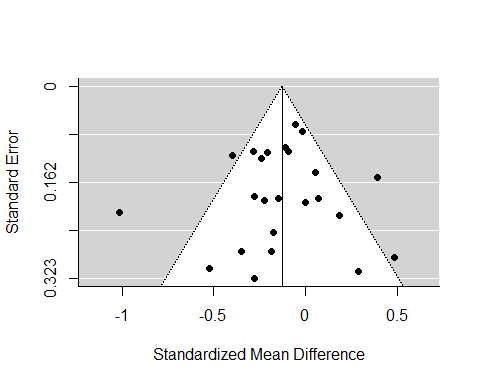


Fig. S3 Baujat plot - DBS vs. ODT PD effects on delayed recall


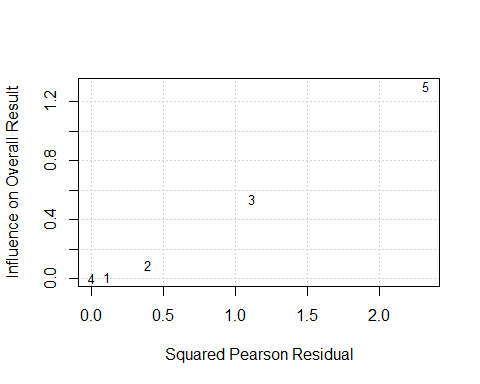


Fig. S4 Funnel plot - DBS vs. ODT PD effects on delayed recall


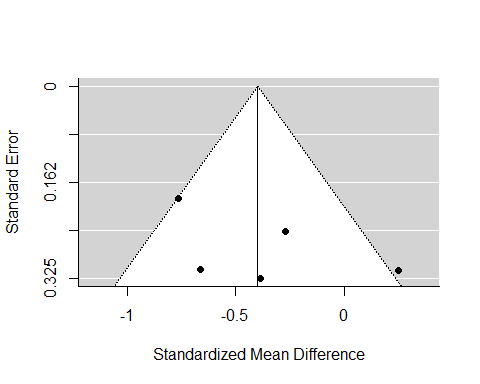


- **Backward Digit Span**

Fig. S5 Baujat plot - DBS effects on backward digit span (overall)





Fig. S6 Funnel plot - DBS effects on backward digit span (overall)





- **Immediate recall**

Fig. S7 Baujat plot - DBS effects on immediate recall (overall)


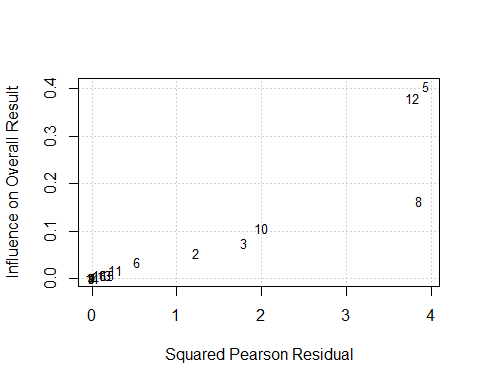


Fig. S8 Funnel plot - DBS effects on immediate recall (overall)


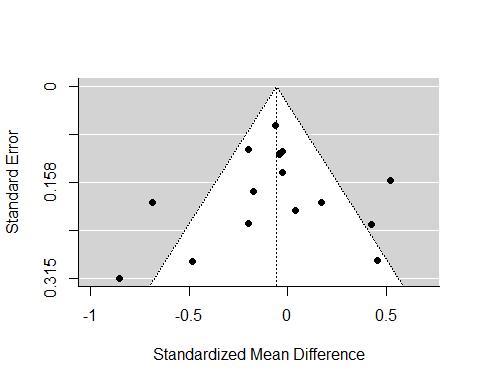


**4.2. EXECUTIVE FUNCTIONS**

- **Phonemic verbal fluency**

Fig. S9 Forest plot – STN DBS effects on phonemic verbal fluency (overall) before Moretti et al. (2003)’s exclusion


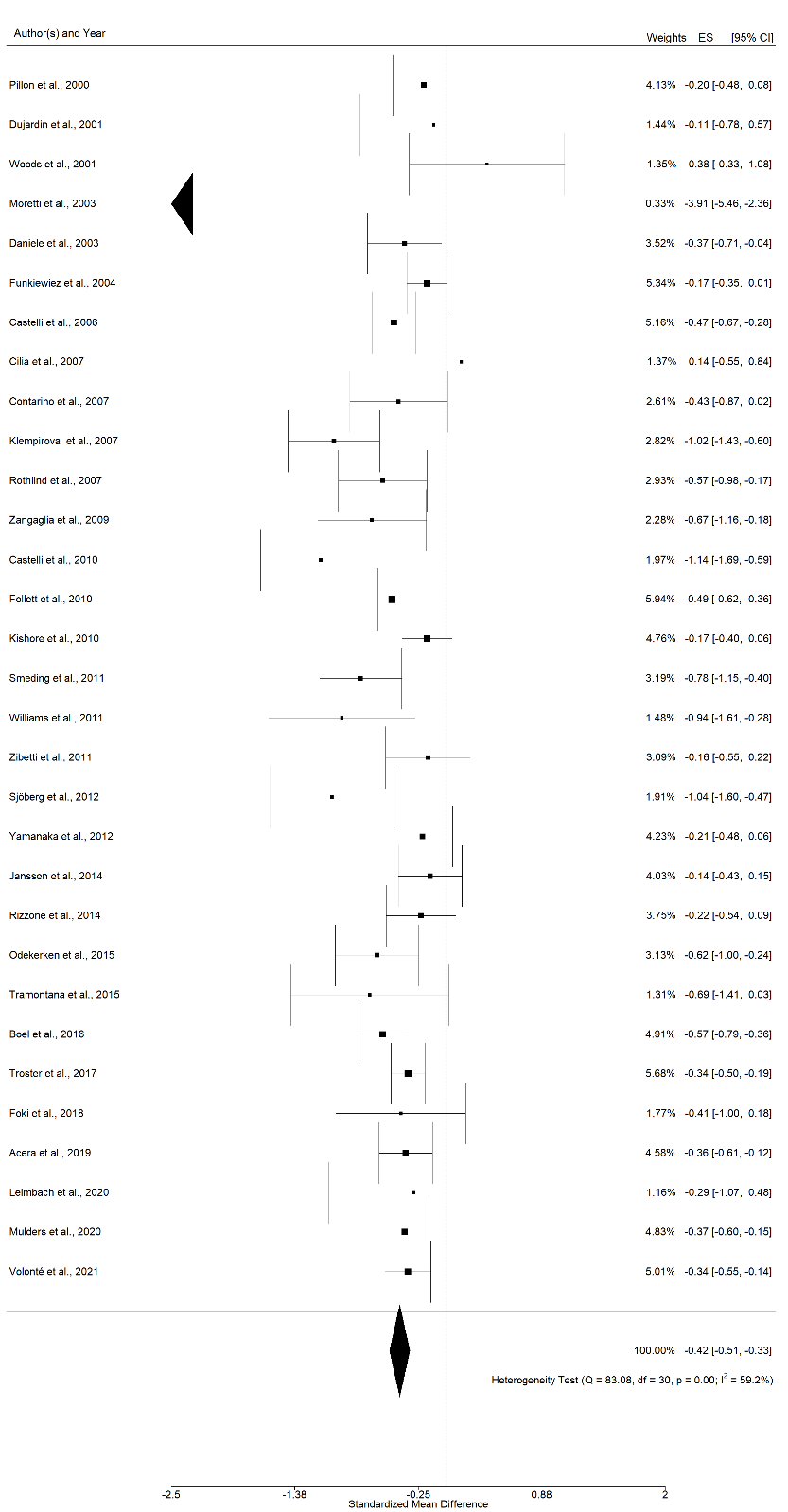


Fig. S10 Baujat plot – STN DBS effects on phonemic verbal fluency (overall) before Moretti et al. (2003)’s exclusion


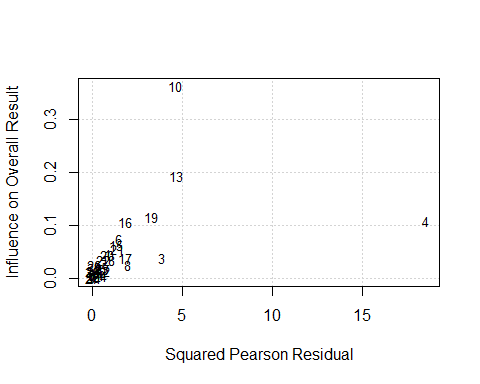


Fig. S11 Funnel plot – STN DBS effects on phonemic verbal fluency (overall) before Moretti et al. (2003)’s exclusion


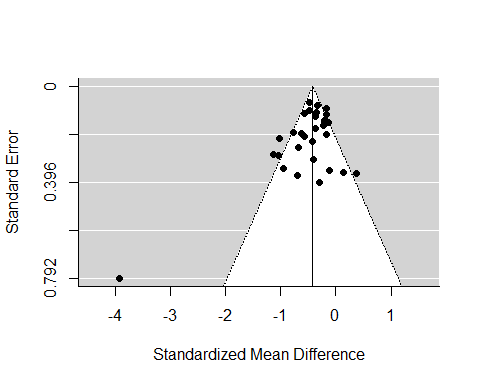


Fig. S12 Baujat plot – GPi DBS effects on phonemic verbal fluency (overall)


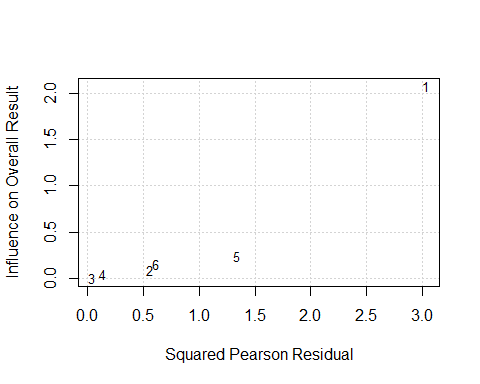


Fig. S13 Funnel plot – GPi DBS effects on phonemic verbal fluency (overall)


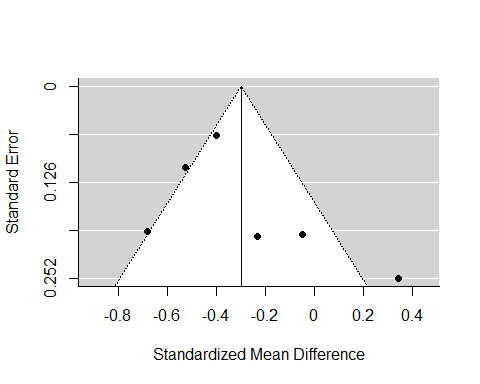


Fig. S14 Baujat plot – DBS vs. ODT PD effects on phonemic verbal fluency


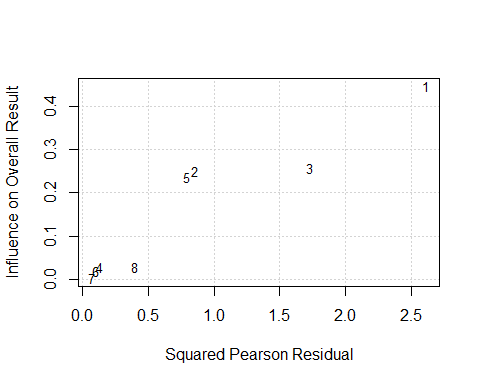


Fig. S15 Funnel plot – DBS vs. ODT PD effects on phonemic verbal fluency


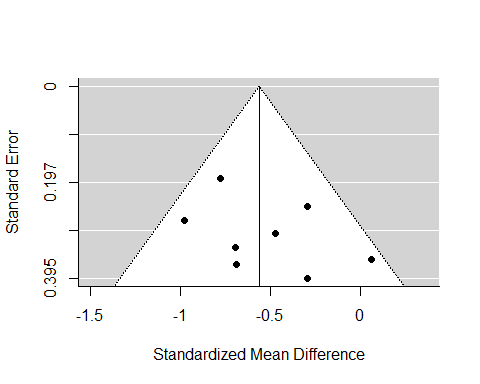


- **Color–word Stroop test**

Fig. S16 Baujat plot – STN DBS effects on Stroop test (color–word) (overall)


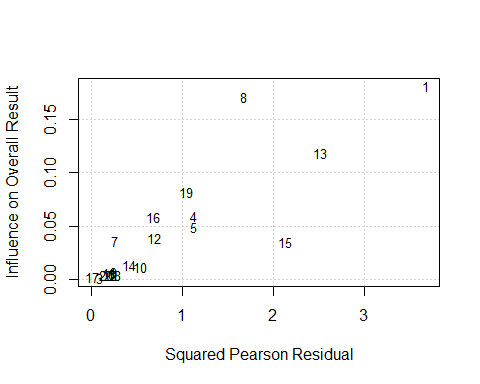


Fig. S17 Funnel plot – STN DBS effects on Stroop test (color–word) (overall)


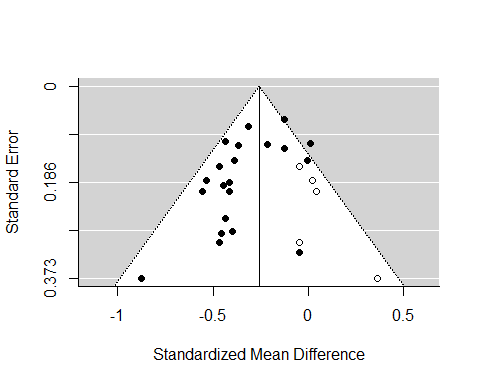


Fig. S18 Baujat plot – GPi DBS effects on Stroop test (color–word) (overall)


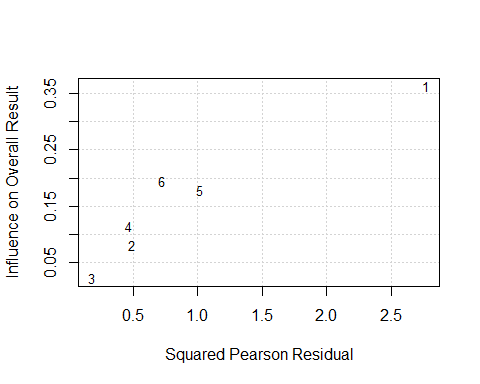


Fig. S19 Funnel plot – GPi DBS effects on Stroop test (color–word) (overall)


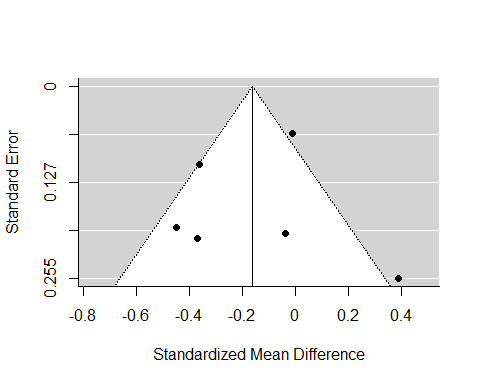


Fig. S20 Baujat plot – DBS vs. ODT PD effects on Stroop test (color–word)


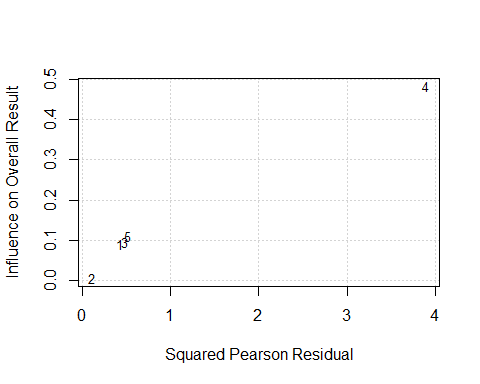


Fig. S21 Funnel plot – DBS vs. ODT PD effects on Stroop test (color–word)

**
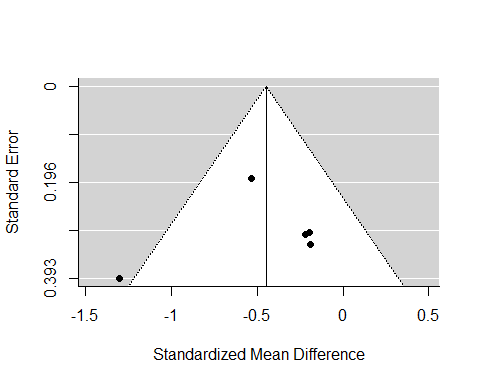
**

**4.3. LANGUAGE**

- **Semantic Fluency**

Fig. S22 Baujat plot – STN DBS effects on semantic verbal fluency (overall)


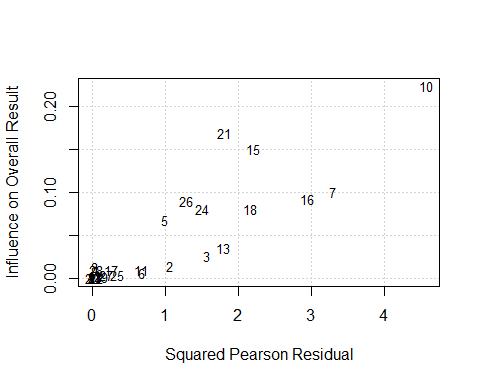


Fig. S23 Funnel plot – STN DBS effects on semantic verbal fluency (overall)


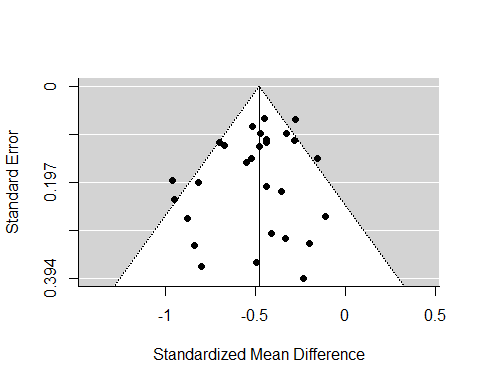


Fig. S24 Baujat plot – GPi DBS effects on semantic verbal fluency (overall)


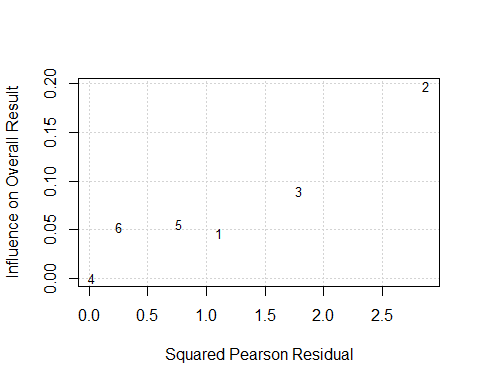


Fig. S25 Funnel plot – GPi DBS effects on semantic verbal fluency (overall)


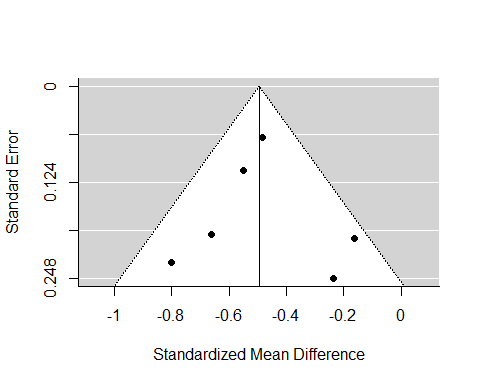


Fig. S26 Baujat plot – DBS vs. ODT PD effects on semantic verbal fluency


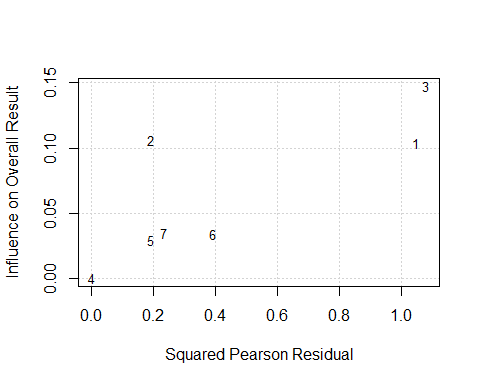


Fig. S27 Funnel plot – DBS vs. ODT PD effects on semantic verbal fluency


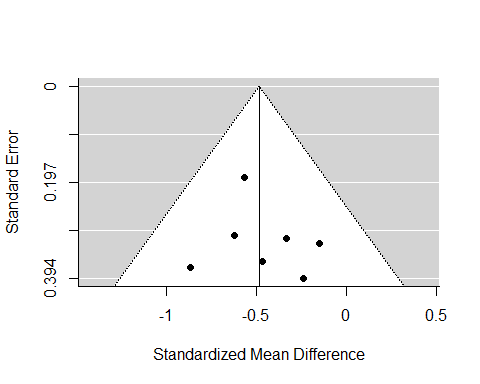


**4.4. DEPRESSION AND ANXIETY**

- **Depression**

Fig. S28 Forest plot – STN DBS effects on depression (overall) before Schuepbach et al. (2013)’s exclusion


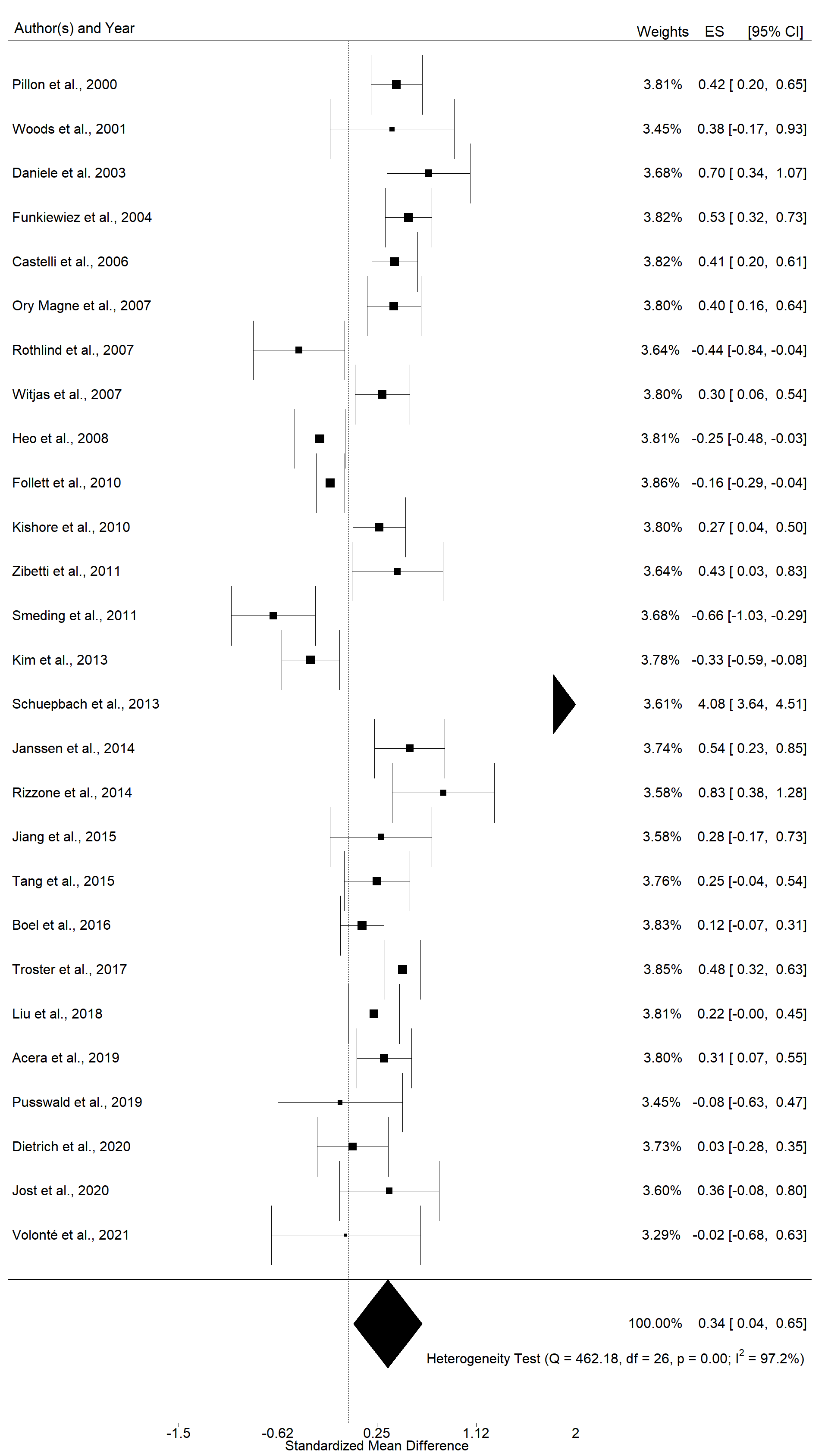


Fig. S29 Baujat plot – STN DBS effects on depression (overall)


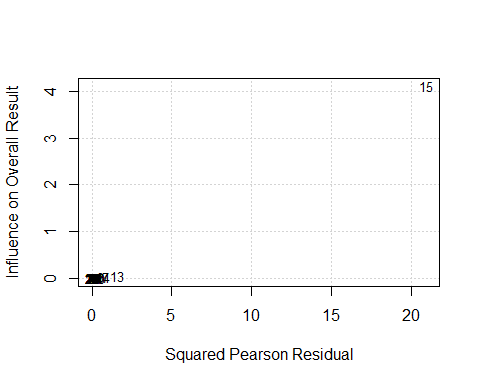


Fig. S30 Funnel plot – STN DBS effects on depression (overall)


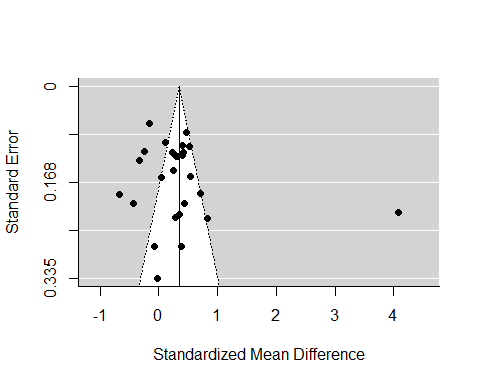


Fig. S31 Baujat plot – GPi DBS effects on depression (overall)


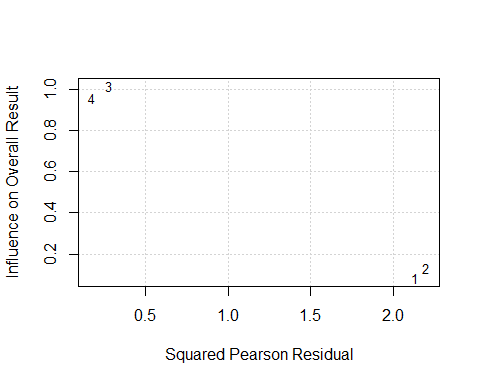


Fig. S32 Funnel plot – GPi DBS effects on depression (overall)


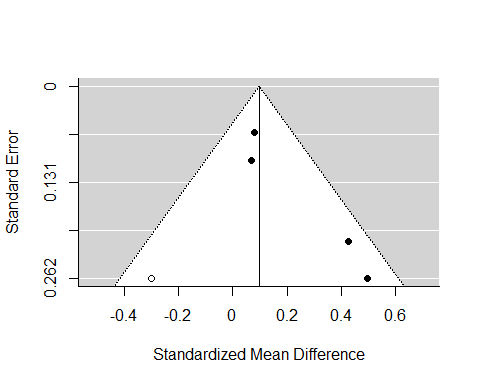


- **Anxiety**

Fig. S33 Baujat plot – STN DBS effects on anxiety (overall)


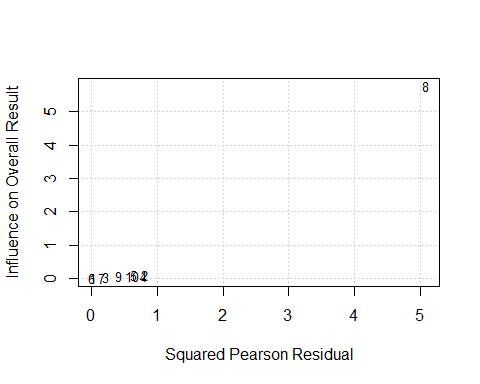


Fig. S34 Funnel plot – STN DBS effects anxiety (overall)


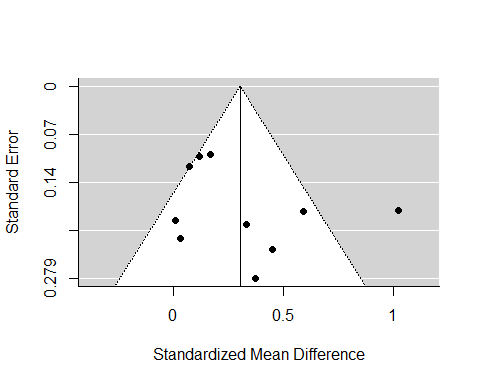

Supplement: Supplementary file 1 — Supplementary file1 (DOCX 585 KB) [file 11065_2022_9540_MOESM1_ESM.docx]
